# A Rapid Review of Ethical and Equity Dimensions in Telerehabilitation for Physiotherapy and Occupational Therapy

**DOI:** 10.3390/ijerph22071091

**Published:** 2025-07-09

**Authors:** Mirella Veras, Jennifer Sigouin, Louis-Pierre Auger, Claudine Auger, Sara Ahmed, Zachary Boychuck, Sabrina Cavallo, Martine Lévesque, Stacey Lovo, William C. Miller, Michelle Nelson, Nahid Norouzi-Gheidari, Jennifer O’Neil, Kadija Perreault, Reg Urbanowski, Lisa Sheehy, Hardeep Singh, Claude Vincent, Rosalie H. Wang, Diana Zidarov, Anne Hudon, Dahlia Kairy

**Affiliations:** 1 Department of Physical Therapy, Rady Faculty of Health Sciences, College of Rehabilitation Sciences, University of Manitoba, Winnipeg, MB R3E 0T6, Canada; mirella.veras@umanitoba.ca; 2 Centre de Recherche Interdisciplinaire en Réadaptation du Montréal Métropolitain (CRIR), Montreal, QC H3S 1M9, Canada; jennifer.sigouin@mail.mcgill.ca (J.S.); louis-pierre.auger@umontreal.ca (L.-P.A.); claudine.auger@umontreal.ca (C.A.); diana.zidarov@umontreal.ca (D.Z.); anne.hudon@umontreal.ca (A.H.); 3 Centre on Aging, University of Manitoba, Winnipeg, MB R3E 0T6, Canada; 4 Faculty of Medicine, School of Physical and Occupational Therapy, McGill University, Montreal, QC H3G 1Y5, Canada; sara.ahmed@mcgill.ca (S.A.); zachary.boychuck@mcgill.ca (Z.B.); 5 École de Readaptation, Faculté de Médecine, Université de Montréal, Montreal, QC H3N 1X7, Canada; sabrina.cavallo@umontreal.ca (S.C.); martine.levesque.4@umontreal.ca (M.L.); 6 School of Rehabilitation Science, University of Saskatchewan, Saskatoon, SK S7N 2Z4, Canada; stacey.lovo@usask.ca; 7 Department of Occupational Science and Occupational Therapy, University of British Columbia, Vancouver, BC V6T 2B5, Canada; bill.miller@ubc.ca; 8 Institute of Health Policy, Management and Evaluation, Dalla Lana School of Public Health, University of Toronto, Toronto, ON M5S 1A1, Canada; michelle.nelson@sinaihealth.ca; 9 Clinical Research Unit, Montreal Neurological Institute/Hospital, Montreal, QC H3A 2B4, Canada; nahid.norouzi@mail.mcgill.ca; 10 École des Sciences de la Réadaptation, Faculté des Sciences de la Santé, University of Ottawa, Ottawa, ON K1S 5S9, Canada; joneil@uottawa.ca; 11 Faculty of Medicine, School of Rehabilitation Sciences, Université Laval, Québec, QC G1V 0A6, Canada; kadija.perreault@fmed.ulaval.ca (K.P.); claude.vincent@rea.ulaval.ca (C.V.); 12 Rady Faculty of Health Sciences, College of Rehabilitation Sciences, University of Manitoba, Winnipeg, MB R3E 0T6, Canada; reg.urbanowski@umanitoba.ca; 13 Bruyère Research Institute, Ottawa, ON K1R 6M1, Canada; lsheehy@bruyere.org (L.S.); rosalie.wang@utoronto.ca (R.H.W.); 14 Department of Occupational Science and Occupational Therapy, University of Toronto, Toronto, ON M5G 1V7, Canada; hardeepk.singh@utoronto.ca

**Keywords:** telehealth, telerehabilitation, e-health, digital health, physiotherapy, occupational therapy, ethics, equity, rapid review

## Abstract

Introduction: The rapid adoption of telerehabilitation in physiotherapy and occupational therapy has transformed healthcare delivery, offering new opportunities for patient-centered care. However, its implementation raises critical ethical and equity-related questions that require proactive strategies to ensure fair and responsible practices. This review examines how ethical disparities and equity-related challenges are reflected in the existing literature on telerehabilitation. Objective: To investigate the presence of ethical-disparity and equity-related aspects in the provision of telerehabilitation in physiotherapy and occupational therapy as reflected in the literature. Data Sources: A rapid review methodology was employed to explore ethical and equity-related challenges in telerehabilitation. The search included articles published in English and French between 2010 and 2023 from the Medline and Embase databases. Study Selection: Articles were selected based on their relevance to ethical and equity considerations in telerehabilitation. A total of 1750 sources were initially identified, with 67 articles meeting the eligibility criteria for inclusion in this review. Data Extraction: Data were extracted based on variables such as age, gender, ethnicity, morbidity, cost, privacy, confidentiality, and autonomy. The data extraction and analysis were guided by the Progress Plus and Metaverse Equitable Rehabilitation Therapy frameworks. Data Synthesis: The findings were analyzed and discussed using a narrative synthesis approach. The results highlighted key ethical considerations, including adverse events, patient autonomy, and privacy issues. Equity-related aspects were examined, access to rehabilitation services and gender considerations. Disparities in technology access, socioeconomic status, and ethnicity were also identified. Conclusions: This rapid review highlights the growing relevance of ethical and equity considerations in the design and delivery of telerehabilitation within physiotherapy and occupational therapy. The findings show inconsistent reporting and limited depth in addressing key domains such as patient autonomy, privacy, and adverse events, alongside disparities related to age, gender, socioeconomic status, and geographic access. Although telerehabilitation holds promise for expanding access, particularly in underserved areas, this potential remains unevenly realized. The review underscores the critical need for structured, equity-driven, and ethically grounded frameworks such as the Metaverse Equitable Rehabilitation THerapy (MERTH) framework to guide future implementation, research, and policy.

## 1. Introduction

Telerehabilitation (TR) has become widely used in the world in the last decade, particularly for reaching geographically remote populations living in disadvantaged situations [1]. TR has been defined as the provision of rehabilitation services through diverse technological approaches, including various rehabilitation tools used for evaluation, assessment, monitoring, prevention, intervention, supervision, education, consultation, and coaching [2]. It has been shown to improve quality of life, maximize functionality, and increase access to rehabilitation services, especially for vulnerable populations, individuals in geographically remote areas, and those with disabilities [3]. Health equity, grounded in human rights principles, refers to the absence of systematic disparities in health or in the key social determinants of health between groups with varying levels of social advantage or disadvantage, such as differences in wealth, power, or status [4]. Ethics is defined here as the study of the nature of morals and the specific moral choices individuals or groups must make, adapted from foundational principles in moral philosophy [5]. In the context of telerehabilitation, this definition encompasses the ethical considerations surrounding patient autonomy, equitable access to care, data privacy, informed consent, and the responsible use of digital technologies in clinical decision-making and remote service delivery.

Many studies have identified challenges and barriers to delivering telerehabilitation services, highlighting concerns that require active strategies to implement and sustain inclusive, ethical, and user-centered rehabilitation practices [6,7]. A scoping review of 22 studies within telemedicine identified four main ethical concerns: confidentiality, accessibility, clinical effectiveness, and the patient-physician relationship [1]. Specific issues arose regarding patient confidentiality. and the accessibility of technology (e.g., absence of reliable high-speed internet, lack of cell phone data plans, lack of smartphones or outdated cell phone models that could not be upgraded to accommodate telemedicine software platforms and video-enabled applications). Moreover, authors reported that patients with sensory impairments (e.g., vision, hearing) or dexterity issues, may encounter difficulties in using smartphones and certain telemedicine platforms [1] These issues appear minor when compared to reports of patients’ experiences with telehealth. For instance, Nittari’s (2020) [8] review on ethical and legal challenges in telemedicine highlighted concerns such as the lack of established protocols in telerehabilitation, perceived intrusiveness of monitoring devices, complexity of payment methods and difficulties accessing consultations and treatments in the case of underserved individuals [8]. Other technical and implementation obstacles highlighted by participants include challenges such as insufficient availability of devices at home, limited bandwidth leading to slow connections, and concerns surrounding the privacy and security of both applications and telecommunication channels [9].

Some studies have specifically identified barriers to the use of technology for older adults, including the use of computers, tablets, internet and e-mail [10]. A telemedicine study on stroke services indicated that usage was highest among younger, male, non-Hispanic white patients, as well as those residing in rural or super rural areas, with “super rural” representing the bottom quartile of rural regions [11]. Even with the rise in telestroke utilization, rural patients continued to have a lower likelihood of receiving IV tPA compared to their urban counterparts [11]. When reviewing the existing literature addressing the intersection of TR and equity indicators, the majority of the cited examples predominantly revolve around the domain of telemedicine, as opposed to rehabilitation.

There is a gap in our understanding of ethics and equity considerations within telerehabilitation. This rapid review aims to summarize evidence from prior reviews and relevant recent studies to contribute to providing recommendations for equitable and ethical telerehabilitation services. Nevertheless, a gap exists in comprehending and summarizing the vast body of literature concerning ethics so that it may be most useful for decision-makers, to orient clinical implementation, and to further research in this area. It is the professional and ethical duty of healthcare system leaders, occupational therapists, and physiotherapists to provide equitable TR access for all patients. Access here is defined as the capacity of individuals to reach and utilize telerehabilitation services, considering technological, geographic, and economic factors.

## 2. Methodology

This rapid review adheres to the guidelines provided in King et al.’s comprehensive guide [12] and aligns with the Cochrane Rapid Reviews-Interim Guidance from the Cochrane Rapid Reviews Method Group [13]. Rapid reviews, as defined by this guidance, represent a form of knowledge synthesis that expedites the traditional systematic review process by streamlining or omitting specific methods. The aim is to produce evidence efficiently for stakeholders while optimizing available resources [13]. This field generates high-volume, rapidly evolving information, which may justify a rapid review. More detailed information on the review methodology is available in the published protocol [4]. Prospero ID: CRD42020207602. The protocol included registering all rapid reviews conducted as part of the larger telerehabilitation synthesis study.

### 2.1. Search Strategy

The search process for this rapid review was a collaborative effort involving a librarian from University of Montreal and research team members. Utilizing the Population, Intervention, Comparator, Outcomes, Timing, Setting, and Study design (PICOTSS) framework, search terms were meticulously developed and used to search in the databases Medline and Embase, chosen for their comprehensive coverage of medical literature relevant to telerehabilitation [14]. The search spanned articles in English and French, including all study designs. Essential keywords, including “telerehabilitation” and related terms like “telehealth” and “online-based intervention” were included in the search strategy to ensure a comprehensive search. The search also extended to terms associated with “physiotherapy” and “occupational therapy,” contributing to a thorough investigation within the telerehabilitation domain.

### 2.2. Eligibility Criteria

This review included review studies published from January 2010 to December 2020 and review articles published between January and December 2023. The limitation to studies published between 2010 and 2020 was applied to capture a foundational body of literature reflecting a decade of telerehabilitation research prior to the COVID-19 pandemic. This timeframe was selected to establish a baseline for understanding how telerehabilitation was implemented and evaluated under routine (pre-pandemic) conditions. To prioritize the most current evidence to inform urgent decision-making, we extended the search to include studies from 2023 to capture more recent literature. The findings from 2023 were broadly consistent with those identified in the 2010–2020 period, reinforcing the stability of key themes and outcomes over time. Eligible studies involved patients of any age who received TR delivered by physiotherapists or occupational therapists, or studies in which these professionals provided the intervention. Interventions encompassed any TR approaches synchronous or asynchronous used to replace or supplement in-person care (e.g., videoconferencing, mobile apps, recorded videos, e-visits, phone calls, wearable devices). All settings were considered, and outcomes had to relate to the International Classification of Functioning, Disability and Health (ICF), technology usability, ethics, patient engagement, satisfaction, feasibility, health service outcomes, or equity-related impacts [15].

Studies were excluded if they involved animal subjects, did not clearly specify whether a physiotherapist or occupational therapist delivered the intervention, or reported outcomes unrelated to the review focus (e.g., diet, weight, blood pressure). Study designs such as protocols, psychometric studies, case reports, or narrative reviews without a systematic search and analysis were excluded. Please find more details on the published protocol [4].

### 2.3. Data Screening and Data Extraction

All identified articles underwent organization and management through Covidence, an online systematic review software [16]. After the completion of database searching, the selected studies underwent screening using the Covidence software to eliminate duplicates. Two authors (M.V. and J.S.) screened all titles and abstracts against the inclusion criteria. Conflicts in the selected abstracts were resolved through discussion (M.V., J.S. and D.K.). The inclusion and exclusion criteria, summarized above and detailed elsewhere [4] were applied. The full-text articles list was screened by M.V. with J.S. and D.K. discussing any questions raised regarding the screening criteria.

Data extraction was conducted using the Covidence systematic review software and Excel capturing key variables such as study country, design, population, telerehabilitation mode of delivering TR services, intervention descriptions, cost, user digital literacy, use of adaptative equipment, technology access, participant autonomy, data security, ethnicity, adverse events, results, limitations, and ethical concerns related to the use of the technology or the conduct of the research.

The data extraction and analysis in this study were guided by the Metaverse Equitable Rehabilitation THerapy (MERTH) framework, which has five critical domains: 1. Quality of care, 2. health services integration, 3. interoperability, 4. global governance, and 5. Humanization [17]. This framework, developed in two phases, was informed by previous frameworks in digital health, the Metaverse, and health equity concerns. The MERTH framework into our methodology ensures a comprehensive analysis of the data, grounded in contemporary perspectives on health and technology. Additionally, the PROGRESS-Plus framework was employed to identify dimensions across which telerehabilitation (TR) inequities may exist [18]. These dimensions encompass place of residence, race/ethnicity/culture/language, occupation, gender or sex, religion, education, socioeconomic status, social capital, age, disability, and sexual orientation [18]. M.V. conducted the data extraction process, with J.O. and D.K. performing extraction from 10% of the included studies. This approach ensures enhanced accuracy and thoroughness the extraction process.

### 2.4. Quality Assessment (Risk of Bias)

We did not use a risk of bias tool for individual studies in our analysis, as our primary aim wasn’t to evaluate intervention effectiveness. Rather, our focus centered on investigating the ethical and equity dimensions of interventions. Nonetheless, we did extract data regarding the overall quality of studies, with particular attention to their inclusion/exclusion criteria and the reporting of ethical and equity considerations. We narrowed our focus to two key questions based on the systematic review appraisal worksheet from the Centre of Evidence-Based Medicine at the University of Oxford: (a) Were all relevant studies adequately included? And (b) Were the criteria used for inclusion/exclusion appropriate, or did they pose a risk of bias? Additionally, we documented whether each review employed a quality assessment method for the included articles.

### 2.5. Data Synthesis

To categorize and summarize extracted data, a narrative synthesis approach was applied to all included studies, including systematic, scoping, rapid, and narrative reviews. This hybrid method facilitated the integration of quantitative and qualitative findings, enabling a comprehensive analysis across varied review types. We organized findings thematically, using a structured framework to examine relationships, summarize key results, and assess consistency across studies [19].

The MERTH [17] and PROGRESS-Plus [18] frameworks guided the synthesis, which supported consistent categorization and interpretation of equity considerations, ethical dimensions, population characteristics, and contextual factors. Quantitative findings (e.g., effect estimates, sample sizes, intervention types) were summarized descriptively, while qualitative data (e.g., patient experiences, feasibility, ethical considerations) were synthesized thematically. Data categories related to equity included access to technology, digital literacy, socioeconomic status, gender, age, geographic location, cost, and education. Ethics-related categories focused on informed consent, privacy and confidentiality and cultural sensitivity.

We employed a pragmatic synthesis approach, which involved organizing findings thematically across the included reviews to identify recurring patterns, ethical and equity considerations, and conceptual gaps. No formal weighting or ranking of studies was applied, given the descriptive and heterogeneous nature of the evidence [17].

#### Analysis of Subgroups or Subsets

In our analysis, we conducted thorough subgroup analyses to discern variations and similarities across disciplines, patient groups, settings, and various telerehabilitation characteristics. By scrutinizing the data within these subgroups, we retrieved information about distinct factors influencing TR practices, allowing for the identification of best practices, potential gaps, and areas for improvement within each discipline. Moreover, our subgroup analysis included individually analyzing physiotherapy and occupational therapy studies. This examination provided a more detailed understanding of the unique dynamics within each discipline and the potential ethical and equity gaps between them.

### 2.6. Changes on the Protocol

Some deviations from the published protocol were made to align with the refined scope of the review. First, we did not apply a formal risk of bias tool, as the primary aim was not to evaluate the effectiveness of interventions. Instead, the focus was examining telerehabilitation interventions’ ethical and equity dimensions. Nevertheless, we extracted data on the overall quality of the included reviews, with particular attention to inclusion and exclusion criteria and reporting ethical and equity considerations.

Another change to the protocol was the decision to limit the synthesis to review articles published up to 2020. As outlined in the search strategy section, we also included additional review articles published between January and December 2023 to enhance the comprehensiveness and relevance of the analysis. This allowed us to incorporate more recent evidence and assess whether findings remained consistent with earlier reviews.

## 3. Results

### 3.1. Search Results and Characteristics of Included Studies

**Figure 1:**
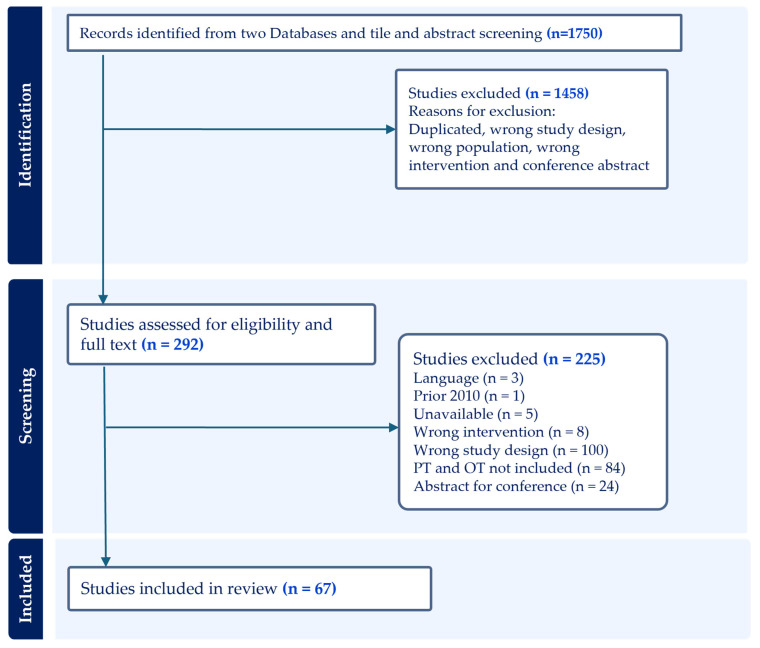
Prisma Flow Diagram of Study Selection Process.

The initial literature search identified 1750 records from Embase and Medline, with 1050 from Embase and 700 from Medline. After removing 14 duplicate articles, a total of 1736 records remained and were imported into Covidence for screening. Two reviewers independently assessed the titles and abstracts of these studies to determine eligibility based on predefined inclusion criteria and methodological relevance. Of these, 1180 articles were excluded for not meeting the inclusion criteria, leaving 556 records for more detailed screening. Following this, 292 full-text articles were assessed for eligibility. Among these, 225 were excluded for various reasons, including publication language, lack of relevance to physiotherapy or occupational therapy, inappropriate study design, or being a conference abstract. Ultimately, 67 studies met the inclusion criteria and were included in the final review. Of these, 16 studies addressed both physiotherapy (PT) and occupational therapy (OT), 42 studies focused exclusively on physiotherapy, and 9 studies focused exclusively on occupational therapy. Further details on the included studies are presented in the Supplementary Material, including Table S1 for studies addressing both PT and OT, Table S2 for exclusive physiotherapy studies, and Table S3 for exclusive occupational therapy studies. The study selection process is outlined in Figure 1 (PRISMA Flow Diagram). 

### 3.2. Study Design

The 67 studies included 35 systematic reviews, 18 systematic reviews with meta-analysis, 9 scoping reviews, and 5 narrative or literature reviews with detailed study descriptions.

### 3.3. Ethics Considerations

**Figure 2:**
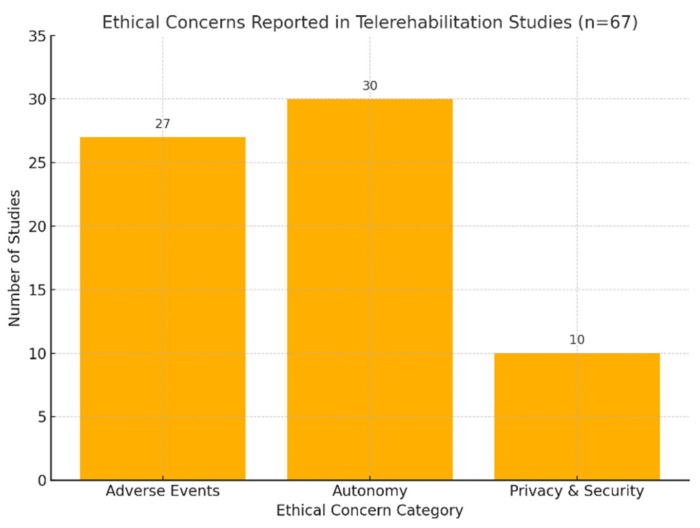
Number of Studies Reporting Ethical Aspects.

This rapid review examined ethical concerns documented across a spectrum of telerehabilitation studies. Among the identified ethical dimensions within telerehabilitation, actual and potential risks for adverse events were mentioned and included as an outcome or comment in 27 review articles [20,21,22,23,24,25,26,27,28,29,30,31,32,33,34,35,36,37,38,39,40,41,42,43,44,45]. Autonomy was another ethical consideration, with 30 studies emphasizing the importance of respecting individuals’ independence and decision-making within telerehabilitation contexts [24,25,26,27,28,30,32,33,34,35,37,39,42,44,46,47,48,49,50,51,52,53,54,55]. Additionally, privacy and security concerns were mentioned in 9 studies, [21,30,33,34,41,43,44,45,56,57] emphasizing the need to protect individuals’ private information during telerehabilitation services. However, studies that did not report specific ethical concerns, highlighting potential gaps or variations in the documentation of ethical dimensions across the reviewed literature (Figure 2). It reflects a major concern that may reflect both limited focus within the included reviews and potential underreporting in the primary studies they summarized. This is notable given the ethical importance of data protection in telerehabilitation and digital health more broadly. It also underscores the importance of standardized reporting frameworks that include digital ethics domains, particularly as telehealth technologies continue to expand in reach and complexity.

In this study, the results regarding ethical concerns were systematically categorized into three primary domains, with adverse events being the foremost reported and mentioned. This category includes safety, harm, potential risk, complications, and unintended consequences. Following adverse events, autonomy is the second most reported or mentioned domain, which includes aspects such as independence, choice, decision-making, self-management and self-determination. Each subcategory is counted within the context of individual studies included in comprehensive reviews or through comments provided by review authors (Figure 2). Lastly, the privacy and security category addresses aspects of confidentiality, security, data protection, information security, and privacy concerns. The number of studies with ethical aspects varied based on the reporting of variables or outcomes, whether they were reported, mentioned, or not reported. Please refer to Table S1 Equity and Ethics Variable or Reporting Across Included Studies for details (Supplementary Material). Table S4 presents the ethics themes and illustrative examples in included studies (Supplementary Material).

### 3.4. Equity Considerations

This rapid review investigated equity aspects across various dimensions within the 67 included studies. Age was reported in 42 reviews containing information on the age demographics of participants [20,22,24,26,28,29,30,32,33,35,36,37,38,39,41,42,43,44,45,46,47,48,49,50,57,58,59,60,61,62,63,64,65,66,67,68,69,70,71,72,73,74,75]. Access to rehabilitation services was another significant focus, with 35 reviews mentioning aspects of TR access [20,21,22,23,30,31,33,34,35,36,37,41,42,43,46,47,48,49,50,52,56,59,60,62,68,70,71,72,73,76,77,78,79,80,81]. Gender variables were reported in 34 reviews [20,22,24,26,29,30,32,36,37,38,39,40,41,42,43,44,45,46,47,50,51,58,59,62,63,69,71,72,74,75,78,80]. Out of the 67 studies included in this review, 25 reviews included at least one study that reported costs, or within the individual studies included, authors commented on the cost of equipment selection [22,24,26,30,32,35,36,38,42,43,44,45,46,52,53,54,57,62,63,65,71,76,79,80,81,82].

**Figure 3:**
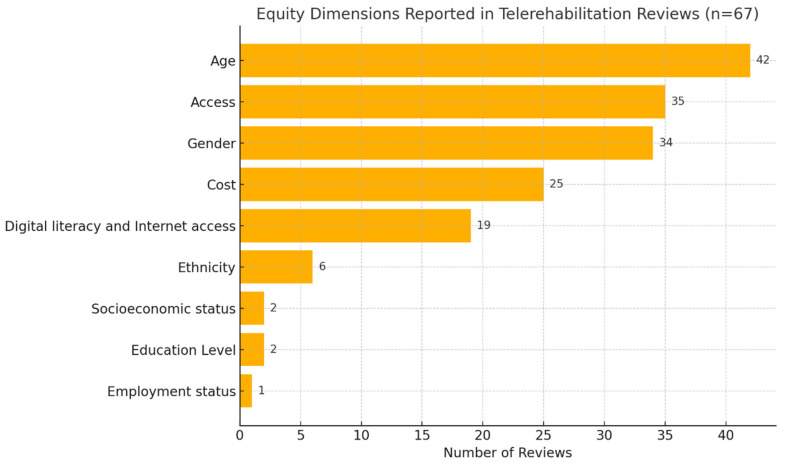
Number of studies reporting equity aspects of the research.

Furthermore, the digital divide in access to technology (internet) and technology literacy were reported in 19 reviews (ten digital literacy comments and nine mentions of access to internet) [21,27,30,31,33,34,39,40,46,47,56,60,70,72,74,76,78,79,80], while socioeconomic status (SES) was reported in 2 reviews [47,83], one review included 9 studies that reported SES [47]. Ethnicity was reported in 6 reviews [24,25,35,47,55,68]. However, education [33,83] and employment status [33] received less reporting, with only 2 and 1 review(s) respectively reporting this equity-related aspects (Figure 3). The limited number of reviews addressing socioeconomic status (n = 2) and ethnicity (n = 6) highlights a critical gap in the current literature. This underrepresentation suggests that telerehabilitation research does not systematically consider key social determinants of health. 

Table S1 describes how various equity aspects of the studies are explored with examples drawn from publications of included studies (Supplementary Material). Each theme corresponds to a specific PROGRESS Plus and MERTH domains, highlighting various dimensions of equity-related concerns in telerehabilitation services. Examples were grouped according to these dimensions to provide a comprehensive understanding of the challenges and factors associated with access, gender, cost, digital divide, socioeconomic status (SES), education, and ethnicity. Access is an equity aspect of the MERTH framework and within the PROGRESS-Plus framework is associated with SES includes factors such as income, education, and occupation. Individuals with lower socioeconomic status may face barriers to accessing healthcare services, including telerehabilitation. The distribution of studies according to equity aspects varied based on the reporting of variable or outcomes, whether they reported, mentioned, or not reported. Please refer to Table S1 equity and ethics variable or outcome reporting across included studies for details (Supplementary Material). Table S5 presents Equity themes and illustrative examples in included studies (Supplementary Material).

### 3.5. Quality Assessment (Risk of Bias)

The critical appraisal of the included studies revealed variability in methodological quality across reviews. Issues such as selection, performance, detection, and attrition bias were frequently identified. Several studies lacked methodological rigour due to small sample sizes, non-randomized designs, and unclear recruitment procedures. Moreover, inadequate reporting of participant characteristics, intervention protocols, and outcome measures limited transparency and interpretability. Age-related bias was also observed, with the underrepresentation or exclusion of older adults from telerehabilitation studies, potentially affecting the generalizability of findings.

Although formal risk of bias appraisal was not a requirement for inclusion/exclusion decisions, we conducted this appraisal to inform our interpretation of the overall strength and limitations of the evidence base, consistent with guidance for narrative synthesis [19] and rapid review methodology [84]. No studies were excluded based on quality alone; methodological appraisal helped contextualize the findings.

Discussion of ethical and equity aspects was integral to our review focus, as these were prespecified outcomes of interest. As such, we examined whether and how studies reported on these domains not as criteria for exclusion but as indicators of relevance to our equity and ethics-oriented review questions. These elements are essential for understanding the inclusivity and accessibility of telerehabilitation interventions.

## 4. Discussion

### 4.1. Ethics Aspects

Examining examples of ethical concerns provided in Table S4, it is evident that adverse events are a significant consideration for TR interventions. For instance, Agostini (2015) [23] emphasizes the need for improved primary research quality to discern the benefits and risks associated with remote rehabilitation carefully. Over 50% of studies did not report any ethical outcomes, as shown in Figure 2. These additions help reinforce the visual data and enhance the integration of quantitative findings into the narrative interpretation Similarly, concerns about image resolution and rapport during telerehabilitation sessions, was highlighted by Mani (2016) [50]. The risk of adverse events resulting from diagnosis errors due to poor image resolution presents significant challenges. Such errors may lead to misinterpretation of musculoskeletal injuries or conditions, potentially resulting in inappropriate treatment approaches or delayed interventions. Moreover, unclear imaging may hinder accurate assessment of telerehabilitation progress, impeding the development of effective therapy plans and prolonging recovery timelines. Failure to detect underlying issues or complications, such as tissue damage or joint instability, poses additional risks, potentially exacerbating existing conditions or causing secondary injuries during TR exercises.

Differentiating between actual adverse events and potential risk were challenging, as it was unclear whether certain studies omitted reporting adverse events or simply did not have any. Additionally, it is important to discern whether adverse events were unreported due to omission by the included studies or because review authors did not mention the adverse events. Without examining the individual articles, drawing definitive conclusions became difficult. Furthermore, discrepancies result from the way in which adverse events were defined across studies, with some adhering to Food and Drug Administration (FDA) guidelines while others lacked clarity on their definitions. For this rapid review, we considered actual reported adverse events and any mentions or comments regarding potential risks as indicators of adverse events. A recent systematic review on the efficacy and safety of telerehabilitation for patients following total knee arthroplasty concluded that existing literature has overlooked patients’ adverse reactions or safety indicators after telerehabilitation. The authors suggested that future studies should incorporate these indicators or outcomes into their assessments for a more comprehensive understanding of telerehabilitation’s safety profile [85]. To address the current lack of consistency in how harms are reported, we recommend that future reviews and primary studies adopt standardized adverse event taxonomies, such as the Common Terminology Criteria for Adverse Events (CTCAE), to differentiate clearly between observed adverse events and potential risks.

In the context of telerehabilitation, respecting patients’ autonomy emerges as an important principle, as highlighted by Chen (2019) [27], who emphasizes participants’ appreciation for flexibility and control over their rehabilitation process. Moreover, research by Mani (2016) [50] and Slattery (2019) [37] underlines how self-management strategies can empower patients, emphasizing the crucial role of autonomy in optimizing outcomes within telerehabilitation settings.

According to Greaney (2020) [86], the concept of self-care as a strategy for managing chronic conditions is gaining attention, with individuals being increasingly responsible for daily health-related tasks [86]. These tasks evolve along a continuum from prevention to active disease management and include medical, behavioral, and emotional aspects. Engaging in self-management can empower individuals and lead to improvements in quality of life, clinical outcomes, and self-efficacy [86]. From an ethical perspective, self-care aligns with established bioethical principles such as autonomy, beneficence, nonmaleficence, and justice. Professional codes of ethics and regulatory guidance also promote personal autonomy in healthcare. Therefore, self-care is seen as a comprehensive strategy for improving patient outcomes and an ethical concept consistent with ethical norms and professional obligations [86].

Despite the well-intentioned premise of self-care and self-management, it can sometimes lead to essential care tasks (e.g., medication management, engage in exercise and drink water) being left undone, resulting in adverse patient outcomes. This critique, primarily based on an interrogation of the ethical principle of autonomy, suggests that self-care may exceed individuals’ capabilities and resources, leading to feelings of abandonment and poor outcomes. Moreover, an emphasis on personal responsibility for health can shift the clinician-patient relationship towards victim-blaming and further marginalize vulnerable individuals and exacerbate health inequalities [86]. The principle of autonomy, increasingly emphasized in healthcare, has roots in individual rights and self-determination but can sometimes conflict with other ethical principles like beneficence and justice. The “autonomy conundrum” arises when vulnerable individuals struggle to exercise autonomy due to their circumstances. This struggle is particularly evident in chronic illness, where autonomy fluctuates between periods of dependence and independence. Higher-order cognitive skills required for self-management may surpass the capabilities of individuals, especially without adequate support [86]. In the context of telerehabilitation, physiotherapists and occupational therapists should carefully consider the complex needs of patients with chronic illnesses. It’s crucial to provide comprehensive support, reporting physical, mental, and social aspects, to facilitate effective self-management and promote autonomy while ensuring patients aren’t overwhelmed. This means tailoring interventions to each individual’s capabilities and context, offering appropriate guidance and resources, and fostering a supportive environment conducive to their overall quality of life and effectiveness of the TR intervention.

Privacy concerns, as exemplified by Pietrzak (2013) [57] and Berton (2020) [33], emphasize the critical need for robust security measures to protect patient data during telerehabilitation sessions. Similarly, in the qualitative study conducted by Guy et al., issues concerning respect for privacy were prominently mentioned by all participants, indicating the universal importance placed on ensuring the confidentiality of patient information in telerehabilitation contexts [87]. The Ethical Necessities and Principles in Telerehabilitation literature review suggests that user agreements should adhere to legal and ethical guidelines to protect patient information. Informed consent, facilitated through electronic forms, is essential to navigating potential ethical issues and informing patients of risks and benefits. However, concerns remain regarding hacking and third-party data ownership, leading to financial losses and compromising patient privacy. Measures such as two-factor authentication should be employed to enhance data security, nevertheless challenges persist in fully protecting medical data despite encryption methods [88]. Furthermore, Iacono (2016) [56] highlights the necessity of involving policymakers and professionals to respond to clinician concerns and ensure the secure delivery of ehealth services.

Therapists can utilize clinical guidelines to guide their telerehabilitation practice, ensuring adherence to evidence-based recommendations and best practices [89]. For example, a clinical guideline on telerehabilitation was developed by a volunteer guideline development group convened by the American Physical Therapy Association. The guideline comprises several recommendations, including guidance regarding risks, harms, costs of implementation, privacy and security [89].

The ethical issues highlighted in this review, including underreported adverse events, fluctuating patient autonomy, and persistent concerns over data privacy, reinforce the critical need for a comprehensive ethical framework such as MERTH. The Equity domain of the MERTH framework responds directly to disparities in care quality and digital access, which may underlie the inconsistent reporting and detection of adverse events in telerehabilitation studies. Similarly, the Health Services Integration domain supports ethically grounded strategies to promote autonomy without overwhelming patients, emphasizing person-centered care planning, shared decision-making, and the provision of adequate self-management support. The tension between autonomy and capability, particularly in patients with chronic illness, is addressed by advocating for tailored, context-sensitive interventions. The Interoperability domain encompasses the ethical imperatives of data security and privacy, highlighting the need for standardized technologies and regulatory alignment to ensure confidentiality in remote rehabilitation settings. The Global Governance domain provides a foundation for establishing accountability and transparency across jurisdictions, especially in the absence of clear adverse event reporting standards. Finally, the Humanization domain underscores the need to sustain therapeutic relationships and maintain patient dignity in virtual environments, countering the risk of depersonalization in TR care.

### 4.2. Equity Aspects

In this review, 31 studies included comments related to access to telerehabilitation or the equipment used for telerehabilitation. Among these, Rochette’s 2013 study [90], which recruited participants from 11 acute care hospitals across urban and rural areas in four Canadian provinces, highlighted the potential benefits of telerehabilitation for individuals in rural and remote areas who lack access to rehabilitation teams or clinicians. The study emphasized that eliminating the need for travel to rehabilitation centers could particularly benefit those with severely restricted mobility or limited access to healthcare professionals, especially in low-resource settings where access to health services is poor but access to devices such as mobile phones is available. Similarly, Laver (2020) [30] emphasized the importance of rapid and remote access to healthcare professionals for tailored support, which has been shown to enhance adherence to Internet-based interventions. Guay (2017) [80] also, suggested that knowing they have access to professional support at any time and place may easy concerns for caregivers. Berton (2020) [33] also highlighted the advantage of telerehabilitation for individuals with severe disabilities, as it eliminates the need for physical travel to rehabilitation centers.

In contrast to the positive aspects highlighted in the previous paragraph regarding the potential benefits of TR, a recent study on the acceptability of telerehabilitation experiences and perceptions by individuals with stroke and caregivers in an early supported discharge program, identified several barriers to the acceptance and use of this approach [91]. Technical issues such as audio configuration problems, the need to relocate devices, adjust camera angles, and lighting concerns were cited as significant challenges. Additionally, the instability of internet connections posed a barrier to effective telerehabilitation sessions. The need for ample space to perform specific interventions, particularly in physiotherapy and occupational therapy, was also considered a constraint. Lack of access to necessary equipment and the absence of human contact and feedback further contributed to the challenges faced in implementing telerehabilitation programs [91]. While technical issues were cited as significant challenges, it’s essential to note that access to technology and technical barriers may not inherently be equity issues. These challenges become equity issues when they disproportionately affect certain demographic groups based on factors like income, race [46], level of ability, or geographic location. For instance, individuals from lower-income households or rural areas may face greater difficulties accessing reliable internet connections or necessary equipment for telerehabilitation sessions.

Telerehabilitation shows promise as an alternative for delivering rehabilitation services [92,93]. However, recent studies have identified barriers that must be acknowledged and addressed to maximize its effectiveness. These include technical issues, unstable internet connections, space constraints, lack of equipment, human contact, and feedback. Strategies to overcome these barriers may involve improving technological infrastructure, providing adequate training and support for patients and healthcare professionals, and enhancing human interaction and feedback within TR platforms. Emphasizing the critical assessment of data collected on variables such as age, gender, income, race, and geographic location is essential for ensuring that telerehabilitation becomes a more accessible and equitable means of delivering rehabilitation services.

Although age was one of the most frequently reported potentially equity-related variables across the reviews, the authors of the included studies generally did not comment on age and its equity implications. Regarding gender, among the studies that did provide gender-related information, there was a lack of discussion regarding the gender implications for telerehabilitation interventions. Such reporting is essential for enhancing the understanding of how telerehabilitation interventions may impact different genders and for informing the development of more inclusive and equitable healthcare practices. Moreover, it is worth noting that many studies reported gender in varying ways, such as numerical counts, means, medians, and percentages, which can pose challenges for making meaningful comparisons across studies. Furthermore, the comments made by certain studies highlight potential gender biases and disparities within telerehabilitation interventions. For instance, Appleby (2019) [32] noted a gender bias towards men, with more male participants than female participants in the study. Differently, Hewitt (2020) [63] observed that most studies reporting on participant gender had a higher number of female participants. Additionally, Yadav (2019) [59] found differences in the receipt of appropriate care between men and women within intervention groups, further emphasizing the importance of considering gender-related factors in telerehabilitation research and practice.

A study investigating gender disparities and access to telehealth services concluded that there is a significant association between telehealth use and gender. The results indicated that male patients exhibited a greater reliance on telehealth services than their female counterparts [94].

A scoping review explored a significant gap in understanding the interplay between sex, gender, and rehabilitation participation and outcomes within health systems. The findings reveal disparities in access, adherence, and outcomes, with women generally experiencing worse outcomes and a higher caregiving burden compared to men [95]. However, the existing literature predominantly focuses on specific rehabilitation types in high-income country contexts, neglecting global geographic and condition-based rehabilitation needs. Moreover, the conflation of sex and gender, along with the misrepresentation of them as binary, further complicates the analysis [95]. Authors of this scoping review suggested that future research should adopt social science and intersectional approaches to comprehensively explore how gender, along with other social norms and structures, influences rehabilitation disparities [95]. Gender is being explored, but we must also consider sexual orientation. LGBTQIA2S+ individuals may face unique challenges in accessing rehab services. Yet, current studies often overlook sexual orientation. Future research should include it for a complete view of disparities. In addition to that, healthcare systems ought to prioritize individualized, gender-sensitive care, ensuring services are tailored to respond to the complex interaction of social norms, roles, and structures to mitigate gender disparities in rehabilitation participation and outcomes across various contexts [95].

Despite the emphasis on the importance of reporting costs in telerehabilitation studies, there still appears to be a lack of comprehensive reporting in this regard [96]. This observation aligns with the findings of Kairy (2009) [7], who emphasized the importance of reporting costs in telerehabilitation studies. Recent studies continue to highlight the limited attention given to the cost-effectiveness ratio perspective in TR [97]. Table S5 presents examples of costs related to equity concerns in telerehabilitation interventions, highlighting various financial aspects associated with ensuring equitable access to TR. Additionally, they mention using low-cost technologies, such as traditional telephone systems, in telerehabilitation for various purposes, including cognitive assessment and assistive device prescription. However, despite the perceived benefits, there is a lack of evidence regarding the cost-effectiveness of TR programs, with studies failing to provide comprehensive data on investment costs or resource utilization. Therefore, addressing this cost-reporting gap is essential for advancing our understanding of the economic implications of telerehabilitation interventions.

The absence of studies investigating differential financial supports, such as providing tablets, funding for apps or devices, or accessibility features based on income status, highlights a significant gap in current research. This limitation underscores the need for further examination and acknowledgment of disparities in financial support provision. Additionally, considering training programs for individuals with low literacy could be beneficial in addressing accessibility barriers.

Telerehabilitation is recognized as a potential solution to bridge access to rehabilitation services gaps; however, there remains a digital divide, particularly among marginalized communities. The digital divide is referred to here as the gap between individuals who have easy access to and proficient use of technology and those who do not [98]. Despite improvements in broadband access, certain demographic groups with chronic conditions and those with lower incomes continue to face barriers to accessing TR services [99]. From a human rights perspective, this gap includes various dimensions, including access to computers and high-speed internet, digital literacy, economic opportunities, and democratic participation. People who lack access to technology may face challenges in engaging with government services, participating in the economy, accessing TR and exercising political rights [98]. Table S5 provides examples of the digital divide, illustrating disparities in access to technology. Moreover, clinicians and patients frequently lack the technical proficiency to navigate information and communication technologies effectively, emphasizing the critical need for comprehensive training and support initiatives. Rehabilitation providers should prioritize addressing technology disparities in research and in clinical practice.

Digital literacy among both clinicians and patients is a critical determinant of equitable TR access and effectiveness. Many individuals, particularly those from low-income, rural, or marginalized communities, face challenges in navigating digital platforms due to limited exposure to or comfort with technology. These barriers can hinder engagement, reduce adherence to rehabilitation protocols, and contribute to poorer outcomes. Clinicians also require ongoing digital literacy development to deliver TR services confidently, interpret data from virtual tools, and support diverse patient needs in technology-enabled environments. Addressing this dual gap requires structured, accessible training programs tailored to varying literacy levels and learning preferences. The MERTH framework underscores the need for equity-oriented capacity building, positioning digital literacy as a foundational enabler of inclusive, accessible, and sustainable TR services. Investing in digital literacy is not only a technical requirement but an ethical imperative to ensure all individuals, regardless of background can fully participate in and benefit from digital rehabilitation innovations. This tension is particularly relevant in telerehabilitation settings involving older adults, especially those recovering from stroke, where cognitive impairment, communication challenges, or fluctuating decision-making capacity may complicate the exercise of autonomy. Tailored consent processes and adaptive interface designs are needed to support autonomy and safety in these populations.

The review highlights substantial equity gaps in current telerehabilitation (TR) literature, particularly in relation to the Equity domain of the Metaverse Equitable Rehabilitation Therapy (MERTH) framework. While many studies referenced access to TR or necessary equipment, few critically examined structural and contextual determinants such as income, race, gender identity, disability, or digital literacy that influence access and engagement. Most research was conducted in urban settings, often omitting rural, remote, and Indigenous populations. Key demographic variables were inconsistently reported and seldom analyzed through an equity lens, with intersectional perspectives notably lacking. M.V. extracted the data and conducted the initial mapping to the MERTH and PROGRESS-Plus frameworks; D.K. and J.S. subsequently reviewed the coding, and any discrepancies were discussed collectively until consensus was reached.

The MERTH framework addresses these deficiencies by embedding equity into all phases of TR development and implementation. It promotes co-design with underserved communities, context-specific adaptations, and prioritization of accessible technologies. Recommendations include subsidized equipment, low-bandwidth applications, and community-based access points. Importantly, MERTH advocates for systematically collecting and analyzing disaggregated equity data to inform inclusive TR policies and practices. Integrating the MERTH framework empowers researchers and practitioners to close equity gaps, reduce digital exclusion, and deliver culturally responsive, ethically sound TR interventions. TR risks reinforcing existing health disparities rather than mitigating them without such structured, equity-centred guidance.

### 4.3. Limitation

The metrics from individual studies were gathered from the included reviews. Each study included in the reviews was not accessed individually. It is possible that some of the included studies were encountered more than once, as they may have been included in multiple review papers. We acknowledge the risk of overlapping studies across reviews. We relied solely on information presented in published reviews, ethical and equity considerations that may have been discussed in the original primary studies could be underreported or omitted, thereby potentially biasing our synthesis toward what reviewers chose to highlight rather than the full scope of ethical reporting in the field

## 5. Conclusions

The findings demonstrated significant variability in how ethical and equity domains are reported in telerehabilitation literature. Ethical concerns such as autonomy, privacy, and adverse events were inconsistently addressed, with limited detail on management strategies. Equity aspects, including access, cost, gender, and digital literacy, were mentioned but often lacked depth and systematic analysis. Although telerehabilitation is frequently framed as a way to improve access for underserved populations, few reviews offered detailed evaluations of actual barriers or facilitators. Reporting on key demographic variables such as socioeconomic status, education, and ethnicity was also limited, constraining assessments of inclusivity. To advance ethical and equitable telerehabilitation, future research must prioritize structured, consistent reporting and engage in collaborative, multidisciplinary efforts to inform inclusive and context-sensitive practice. Future studies should prioritize disaggregated data reporting and the use of equity-oriented frameworks to capture how race, income, and other intersecting factors shape access to and outcomes of digital health interventions. Incorporating these dimensions is essential to ensuring that telerehabilitation approaches are not only effective but also equitable across diverse populations.

## Supplementary Materials

The following supporting information can be downloaded at: https://www.mdpi.com/article/10.3390/ijerph22071091/s1, Table S1: Occupational therapy and physiotherapy study characteristics (N = 16). Table S2: Physiotherapy study characteristics (N = 42). Table S3: Occupational therapy study characteristics (N = 9). Table S4: Ethics Themes and Illustrative Examples in Included Studies. Table S5: Equity themes and illustrative examples in included studies.
