# Supplementary material for: A Rapid Review of Ethical and Equity Dimensions in Telerehabilitation for Physiotherapy and Occupational Therapy
Source: Int J Environ Res Public Health. 2025 Jul 9;22(7):1091. doi: 10.3390/ijerph22071091 (PMC12294586; doi:10.3390/ijerph22071091)
Supplement: Supplementary file 1 [file ijerph-22-01091-s001.zip › Table S1- Occupational therapy and physiotherapy study characteristics (N16) Apr 02 2025.pdf]

Table S1: Occupational therapy and physiotherapy study characteristics (N=16)

Study Author<br>Year<br>Study Design<br>N# included studies	Participants<br>Age<br>Sex<br>Morbidity/Population	Outcomes<br>ICF Domains	Key findings	Ethical principles	Equity aspects
**Agostini 2015<br>SR and MA<br>N=12**	Stroke, cardiac patients, TBI, MS, TKA, SCI<br>**Age and Sex:** Not reported	Body functions and structures, activities and participation	The effectiveness of telerehabilitation in improving motor function, irrespective of the underlying condition, remains inconclusive based on the available evidence. However, there is compelling evidence indicating a significant positive impact on patients who have undergone orthopaedic surgery. This suggests that the heightened level of rehabilitation intensity offered by telerehabilitation holds great promise as a viable treatment option for patients.	**Adverse event**<br><br>"The current data are encouraging and support continuity of rehabilitation care through ICTs, but the quality of primary research has to be improved dramatically to have a clearer picture of benefits and risks associated with assisting patients at a distance, once discharged at home."	**Cost**<br><br>It is essential to avoid resource wastage and inconclusive findings from primary research. Future trials on telerehabilitation should incorporate cost accountability and cost-effectiveness analyses alongside clinical outcomes.<br><br>**Access**<br><br>"The conclusions from the above evidence suggest that telerehabilitation offers an opportunity for equitable access to rehabilitation services for individuals living in remote areas or unable to reach local health providers because of physical impairments." (authors' comment)
**Amatya 2015**	MS	Body functions and structures,	There is growing awareness of telerehabilitation as a	**Adverse events:**<br>None of the included studies	**Comments on costs:** There was no evidence of these

**SR**<br>**N=12**	**Age:** mean 39.7 to 52.5<br>**Sex:** more than 50% of females	activities and participation, personal factors	management option for pwMS. Although high-quality studies on its effectiveness are lacking, existing evidence supports its use for pwMS. Telerehabilitation programs have shown benefits, but findings are inconclusive due to program diversity. These conclusions are tentative, and the research gap should not be taken as evidence of ineffectiveness in pwMS interventions.	reported any serious adverse effects attributable to telerehabilitation.<br><br>**Autonomy:** Significant improvement in Self-Efficacy for Managing Fatigue Scale, self-reported physical activity, self-management, daily self-performed sessions (? Question for Anne)	programmes' cost-effectiveness. Costs and access associated with travel and limited access to services (minimizing the barriers of distance, time, cost and healthcare system load). No studies reported any data on cost-effectiveness, investment costs or resource utilization.<br><br>**Ethnicity:** three studies reported ethnicity (19 Caucasian,<br>95.6% Caucasian and<br>7% African Americans)<br>No comments on the ethnicity (re-check the paper). No further discussion or comments regarding the ethnicity of participants in these studies.
**Amatya 2019**<br>**SR**<br>**N=15**	MS<br>**Age and Sex** not reported	Body functions and structures, activities and participation, personal factors and environmental factors	Regular specialist evaluation and follow-up to assess the rehabilitation needs of individuals with various types of MS can be beneficial, although the certainty of evidence varies among different interventions. Structured, multidisciplinary rehabilitation programs and physical therapy (exercise or physical activities) can enhance functional outcomes and quality of life. However, the evidence for many rehabilitation interventions should be viewed cautiously because most reviews did not include data from recent studies.	**Adverse events:**<br>Authors defined serious event according to FDA. Exercise therapy appears safe and does not increase the risk of MS relapse or adverse events. It can reduce self-reported fatigue for people with MS and can be prescribed without harm. However, there's no clear evidence regarding the best type, duration, intensity, or frequency of exercise due to study diversity. Overall, exercise therapy is beneficial	**Comments on costs:**<br>Despite a lack of high-quality evidence, various telerehabilitation interventions can be a cost-effective way to provide rehabilitation in convenient and remote settings where services are limited. However, included reviews did not offer details on optimal therapy settings, type, intensity, duration, or cost-effectiveness. No convincing evidence supports the cost-effectiveness of

			To address these knowledge gaps, more studies with appropriate design, reporting on modality type, intensity, and cost-effectiveness, are necessary.	for pwMS not in the middle of an exacerbation.<br><br>**Autonomy:**<br>Three studies included outcomes of self-efficacy, self-care and self-reported fatigue	multidisciplinary programs or the ideal therapy "dose." Given rising healthcare costs and growing demand for rehabilitation services for people with MS, it's essential to justify the expense of these services.
**Chen 2015 SR and MA N=11**	Stroke<br>**Age:** median: 66 Mean: 53 to 75.6<br>**Sex:** men: 18% to 100%	Body functions and structures, activities and participation, personal factors	This review offers limited evidence that telerehabilitation is as effective as conventional rehabilitation in enhancing the abilities of daily living and motor function in stroke survivors. Urgent research, particularly randomized controlled trials in the field of telemedicine rehabilitation, is needed to expand the evidence base.	**Adverse events:** Out of the 11 studies included, none provided information on cognitive function or adverse events.<br><br>**Autonomy:** two studies reported Self-care and self-Esteem Scale outcomes.	**Cost:** Only one study reported cost-effectiveness data. In this study, they calculated the costs for human resources, roundtrips to the hospital, and equipment for both the telerehabilitation and control groups. The study found that the overall expenses for a participant in the telerehabilitation group were $654.72 lower than those in the conventional clinical rehabilitation group in their scenario.
**Chen 2019 SR N=31**	Stroke<br>**Age and Sex:** Not reported	Body functions and structures, activities and participation and personal factors	The review highlights that the technologies described in the included studies have the potential to improve patients' motor skills, offer quality rehabilitation similar to conventional methods, enhance daily activities, provide a sense of control, and offer the convenience of home-based rehabilitation. However, the main challenges in implementing these technologies involve insufficient consideration of complex home environment factors. The study suggests that technology design should focus	**Autonomy:**<br><br>“Participants reported a sense of control over their rehabilitation and appreciated the flexibility of scheduling the day and time to exercise. Participants demonstrated significant increase in self-management and functional measures and reduction in time on tasks.”<br><br>**Comment on patient’s challenges:**	**Cost:**<br>“Tele-rehabilitation has the potential to reduce the duration of inpatient hospitalization by helping patients conduct rehabilitation at home, and thus reducing cost.”<br><br>**Digital literacy**<br><br>“Qualitative findings also reveal two practical factors to consider in designing home-based rehabilitation technologies: patients’

			on engagement, incorporating external and internal motivation, and addressing home environment aspects, such as social context, practical challenges, and technical barriers.	"It is crucial to consider the practical challenges of patients and their families, e.g., time management and space requirement. Time management has been reported as a concern for patients who are assigned rehabilitation therapy sessions daily, e.g., five days per week."<br><br>**Pain and fatigue:**<br>"Patients occasionally reported difficulties with fatigue and physical pain and found it more difficult to engage with the game when they are tired."<br><br>**Environmental challenges:**<br>"Besides time and life factors, studies also suggest considering the requirements of physical space, such as space requirements, placement of furniture, lighting condition."<br><br>Comments from authors:<br>"Studies suggested the im-portance of selecting a place for training, such as placement of furniture and lighting condition. Sometimes the size and placement of the rehabilitation system made it difficult for some patients and their family members to	physical space and technical proficiency."<br><br>"Patients and therapists also experienced frequently encountered technical problems, such as using a motion tracking system, following the necessary steps to shut down a system, and charging the system battery. Particularly, it is most crucial to provide technical support in getting started with the system. Some patients needed technical assistance from family members at home or therapists over telephone. In some projects, researchers have considered the potential technical issues and intentionally spent more time in the sessions in the first week to avoid technical issues. Home-based technologies generally require a patient to operate a system without help. Therefore, it is imperative that patients are able to use a rehabilitation system without technical barriers. Helping patients overcome technical barriers is emphasized in a number of projects. Particularly, it is most

				move around the home. Patients might discontinue using the systems that take too much space in the home."<br><br>**Ethical Considerations in Home-Based Rehabilitation: Prioritizing Family and Social Support:**<br><br>"For example, one study emphasized the role of care-givers in stroke recovery, while another, using the Ball Funnel rehabilitation system, allowed the patient to play games with her son. A case study indicated that incorporating activities that allow participants to compete with family members might facilitate compliance and reduce patients' social isolation. Therefore, it is necessary to consider the family and social environment when patients conduct therapy exercises at home."	crucial to provide technical support in getting started with the system."
**Davila Castrodad 2019 Narrative review N=20**	Knee arthroplast y<br>**Age:** one study reported age 50–85 years	Body functions and structures, activities and participation	This systematic review focused on postoperative rehabilitation modalities and their current state of consensus, duration, intensity, and delivery. The study analyzed rehabilitation protocols from the past five years and found that continuous passive motion and	**Adverse event**<br><br>Bade et al study: adverse event rates: Hight intensity Exercises (1 fall) vs. Low intensity exercises (3 falls); P=0.78	**Costs**<br>"Despite the investment, telerehabilitation is especially notable as it may afford longer-term reductions in costs to healthcare systems. Given that post-discharge care

	**Sex:** Not provided		inpatient rehabilitation may not offer additional benefits to patients or the healthcare system. On the other hand, early rehabilitation, telerehabilitation, outpatient therapy, high-intensity and high-velocity exercises appear to be effective forms of rehabilitation. Furthermore, weight-bearing biofeedback, neuromuscular electrical stimulation, and balance control are beneficial additions to conventional rehabilitation. It was noted that some studies lacked clear descriptions of conventional rehabilitation protocols, the duration of therapy sessions, and their timing. The review emphasizes the need for future studies to provide detailed descriptions of their methodologies to support high-quality assessments and the development of standardized protocols.	Han et al. study: HEP and usual care adverse events: 9% vs.7% readmissions; P>0.05 for all	costs can account for at least 36% of the episode of care for joint arthroplasty, healthcare savings secondary to the implementation of telerehabilitation or web-based therapy can have a significant impact. Typical 6-week driving restrictions require TKA patients to rely on others for travel, but these home-based programs seem to mitigate this matter. Despite potential cost reductions with the implementation of both telerehabilitation and home-based therapies, the caveat with home programs is the lack of start-up costs associated with telecommunication technologies.Thorough cost analyses have shown that inpatient therapy substantially increases costs. Hence, given the high cost differential and no difference in outcomes, inpatient therapy looks to be an expensive intervention that does not provide additional benefit. Although these machines are costly, their utility in postoperative rehabilitation may be effective.” (comemnts)
**Iacono 2016 Scoping Review**	Parkinson’s Disease, Cystic	Not reported	The full potential of eHealth in the field of allied health has yet to be realized. While research has	**Technologies barriers**	**Cost**

**N=44**	Fibrosis, orthopedic issues (e.g., Knee, shoulder and elbow problems).<br><br>**Age and Sex**<br>Not reported		shown that traditional assessment and intervention services in allied health can be effectively implemented through eHealth, there is room for more innovative approaches. To harness the true benefits, it is crucial to address concerns voiced by clinicians and demonstrate novel service delivery methods. One way to achieve this is by engaging stakeholders from the communities that could benefit from eHealth and employing comprehensive research strategies. This shift in research focus towards pioneering practices has the potential to bring about significant advantages for both service providers and recipients.	"In a study involving a podiatry clinic providing chronic wound care in rural Western Australia, barriers to ehealth included delays in installing software and losing staff trained in the technologies because of staff turn-over."<br><br>**Handling Data/Data protection/Security (Maintaining anonymity and confidentiality while handling data)**<br>" Further research is needed into information privacy, how ehealth supports continuity of care and promotion of client-clinician relationships. There is also a need to involve Information communication technologies (ICT) policy makers and professionals, and others involved in ehealth implementation to determine the veracity of clinician concerns about lack of support and resources and harness the evolution of ICT to facilitate ehealth delivery to meet varied needs."	"There is a need to address clinician concerns about whether benefits, in terms of professional collaboration, continuity of care and practice efficiency, outweigh costs and perceived barriers, including risks to patient privacy, productivity and patient-practitioner relationships."<br><br>"Ehealth, the use of information communication technologies (ICT) for health service delivery, offers the potential to improve efficiencies and quality in health care delivery in the face of increasing costs and skilled health workforce shortages."<br><br>**Access rural area**<br><br>"Pearce et al. study demonstrated the feasibility of tele-health delivery of audiology services to patients located in rural and remote locations."<br><br>"A high level of access to the internet amongst Australians demonstrates a vector through which the benefits of ehealth for rural and remote areas can be realised, particularly assisted by the National Broadband

					Network (NBN). Unfortunately, the specific mechanisms and nature of the infrastructure have been disputed across governments, resulting in delays in the roll out and inequalities in access."<br><br>"Advantages, such as saving time spent travelling to clients can provide motivation for extending the benefits of ehealth, particularly for rural clients, providing the potential for more frequent contact and support from clinicians. Researching such initiatives would seem a logical next step, as well as into the client experience of ehealth in the con-text of variable speed of internet access across rural and remote Australia."<br><br>"In the only direct trial study involving more than one allied health discipline, Crotty and colleagues [53] evaluated the feasibility of telerehabilitation, provided largely by speech pathologists and physiotherapists, for community rehabilitation and rural nursing home patients. Descriptive data

					indicated good outcomes for most patients in terms of achieving rehabilitation goals, and acceptance by clients as well as clinicians, who also benefited from reduced time in travel. This study was notable for the use of off-the-shelf technology, such as tablet devices, rather than dedicated telerehabilitation equipment as used in many studies."<br><br>**Access/Environment barriers**<br>"Additional factors influencing clinician adoption of ICT may include their access to the required equipment within the work setting, as well as system-level supports (commitment of managers, investment of the organisation) as much as attitude of the practitioner."
**Khan 2015**<br>**SR**<br>**N=9**	MS<br>**Age**<br>Mean age of participant s varied from 41 to 52 years (mean 46.5 years)<br>**Sex**<br>The majority of the	Body functions and structures, activities and participation and personal factors	"There is currently limited evidence on the efficacy of telerehabilitation in improving functional activities, fatigue and quality of life in adults with MS. A range of telerehabilitation interventions might be an alternative method of delivering services in MS populations. There is insufficient evidence to support on what types of telerehabilitation interventions are effective, and in which setting. More robust trials are needed to build evidence for	**Adverse event**<br><br>No studies reported any serious harm from telerehabilitation.<br><br>No serious adverse events reported.	**Cost**<br><br>There was no information on the associated costs.<br><br>Cost-effectiveness Not measured in any of the studies.

	participants were women (proportion ranging from 56% to 87%, mean 74%)		the clinical and cost effectiveness of these interventions."		
**Laver 2020 SR N=22**	Stroke<br>**Age**<br>Most participants in the included studies were aged in their 50s,60s, and 70s<br><br>**Sex**<br>Similar numbers of men and women were included, with the exception of two studies (Chumbler 2012; Smith 2012), for which only men were recruited.	Body functions and structures, activities and participation and personal factors	Although there is a growing number of randomized controlled trials (RCTs) assessing the effectiveness of telerehabilitation, it remains challenging to draw firm conclusions due to substantial variations in interventions and comparators across studies. Furthermore, many of the studies included in this review lacked adequate statistical power and were at risk of bias. Currently, there is limited to moderate-level evidence regarding whether telerehabilitation is a more effective or equally effective means of providing rehabilitation. Short-term telerehabilitation programs following hospital discharge have not shown significant improvements in reducing depressive symptoms, enhancing quality of life, or promoting independence in daily activities when compared to standard care.<br>Comparative studies between telerehabilitation and in-person therapy have generally yielded similar outcomes, suggesting that telerehabilitation is not inferior.	**Adverse event**<br><br>"The use of technology to facilitate communication may lead to miscommunication. For example, the healthcare professional may make errors in assessment of the patient, or the patient may misunderstand advice or instructions provided by the healthcare professional. We were unable to identify any information in the included trials regarding harms associated with telerehabilitation, although one study reported that non-serious adverse events had occurred due to pain and fatigue."<br><br>**Autonomy**<br><br>"Participants received the same stroke education as the control group with an additional 3 telephone follow-up calls at 1 week,	**Cost**<br><br>"No studies included in our original review reported information about the cost-effectiveness of telerehabilitation (Laver 2013). In this updated version of the review, four studies reported information about treatment costs or service utilisation, or both (Bishop 2014; Llorens 2015; Rochette 2013; Saal 2015). Bishop 2014 found that there was a significant reduction in visits to the doctor (for the stroke survivor and caregiver combined) in those receiving the intervention at the three-month follow-up assessment but this was not sustained at six months. Rochette 2013 found no significant differences between groups in unplanned use of health service, and Saal 2015 found no significant differences between groups in relation to medical care (over 3 months) or health service

			Some studies have indicated that telerehabilitation may offer cost savings, but information on cost-effectiveness is lacking. Only two trials reported on the occurrence of adverse events, and they found no serious adverse events associated with telerehabilitation. However, the field of telerehabilitation is still evolving, and further research is necessary to establish more conclusive findings. Additionally, while this review primarily assessed the efficacy of telerehabilitation through RCTs, studies employing mixed methods to assess the acceptability and feasibility of telehealth interventions are highly valuable for evaluating outcomes.	and at 1 and 3 months after discharge,<br>each lasting 15 to 20 minutes, to promote self-management techniques and maintenance of behavioural improvements."<br><br>**Handling Data/Data protection/Security (Maintaining anonymity and confidentiality while handling data)**<br><br>"Studies rarely reported on these factors or how investigators dealt with issues of privacy and protection of data."	use (over 12 months).<br>Llorens 2015 calculated the cost of the telerehabilitation intervention per participant to be $835.61compared to $1490.23 for the in-clinic programme, therefore, the diference in cost between the interventions was $654.72 per participant."<br><br>"Furthermore, although telerehabilitation is purported to reduce the cost of administering an intervention, none of the studies included in this review reported on cost-effectiveness. Randomised trials are now beginning to describe the costs of telerehabilitation and compare these costs to the more expensive in-person model of service delivery. Studies are also examining effect on health service utilisation following intervention, although there is currently insufficient evidence upon which to draw conclusions."<br><br>"However, establishment of telerehabilitation services may be expensive because of the costs of equipment, training, and ongoing technical support. Therefore, it is important to determine

					whether telerehabilitation services once established.”<br><br>“Furthermore, the use of telerehabilitation may resulting cost savings in various ways. Reduced travel time (for clinicians who visit patients in their own home) may mean that clinicians are able to fit more consultations into a single day. “<br><br>“However, establishment of telerehabilitation services may be expensive because of the costs of equipment, training, and ongoing technical support. Therefore, it is important to determine whether telerehabilitation services once established may result in the desired outcomes. The potential advantages of telerehabilitation are clear and have the potential to facilitate access to services (thereby improving equity) and reduce costs associated with providing rehabilitation programmes.”<br><br>**Access**<br><br>Rochette 2013 study Recruited from 11 acute care hospitals located in urban

					and rural areas across 4 Canadian province.<br>"People in rural and remote areas are unlikely to have access to rehabilitation teams with expertise in stroke, and they may not have access to rehabilitation clinicians at all. Eliminating the need for travel to rehabilitation centres may also benefit people with severely restricted mobility who have difficulty travelling or are unable to travel. Telerehabilitation is also likely to be beneficial in low-resource settings where access to health professionals is poor but access to devices such as mobile phones is present." (authors' comment)<br><br>**Internet access**<br><br>"More information is needed regarding the support required to administer telerehabilitation: whether a caregiver is required to assist, how much technology support is required, and whether the person needs to have a certain infrastructure in place (such as a high-speed Internet connection)."<br><br>**Digital Literacy**

					Clinicians and patients may not possess the technical expertise to establish systems and to troubleshoot information and communication technologies. It has been recommended that service providers ensure that technical requirements are met(such as having adequate bandwidth), provide access to technical support and provide training to all users (clinicians and patients).Concerns have also been raised about the security of data transfer and how patient confidentiality can be maintained (American Telemedicine Association 2010)."
**Rintala 2018 SR and MA N=13**	Stroke<br><br>**Age**<br>Mean = 65<br><br>**Sex**<br>Majority of participant s were male	Body functions and structures, activities and participation and personal factors	This systematic review indicates that technology-based distance physical rehabilitation shows comparable effectiveness to traditional treatments in enhancing activities of daily living (ADL), upper and lower extremity functioning, balance, physical activity, and participation among individuals with stroke. However, contradictory findings arose regarding walking. To validate the efficacy of technology-based distance physical rehabilitation interventions in enhancing the	Not reported	**Cost**<br><br>"Future studies should also focus on the resource utilization and cost-effectiveness of technology-based distance physical rehabilitation interventions compared with traditional or similar treatments." (author's comments)<br><br>"To understand the benefits of using technology in physical rehabilitation interventions, one must

			physical functioning of stroke survivors, additional research is warranted.		understand its benefits in terms of resource utilization and cost-effectiveness. Unfortunately, our systematic review did not observe any indication of these approaches in the included studies, which was consistent with that observed in previous similar systematic reviews. These aspects are crucial for understanding whether technology-driven distance rehabilitation interventions are beneficial for the healthcare system without overlooking the meaningful and goal-orientated rehabilitation of persons with stroke. Therefore, future studies should also focus on the resource utilization and cost-effectiveness of technology-based distance physical rehabilitation interventions compared with traditional or similar treatment.” (author’s comments)
**Sarfo 2018 SR N=22**	Stroke<br><br>**Sex and age**<br><br>Not reported	Body functions and structures, activities and participation, personal factors and environmental factors	“Overall, tele-rehabilitation interventions were associated with significant improvements in recovery from motor deficits, higher cortical dysfunction and depression in the intervention groups in all studies assessed but significant differences between intervention versus control groups were reported in 8 out of 22	Not reported	**Cost**<br><br>“The study by Lloréns R et al which assessed cost-effectiveness of tele-rehabilitation showed that the in-clinic intervention resulted in more expenses than the telerehabilitation

			studies in favor of tele-rehabilitation group while the remaining studies reported non-significant differences."		program (654.72 $ per person)."
**Yadav 2019 SR and MA N=15**	Post fracture<br><br>**Age**<br>Majumdar et al. (2008): Median 60y; Jaglal et al. (2012): mean 66y; Bedra et al. (2015): mean 77y; Dickson et al. (2007): mean 56.5 and Roux et al. (2013): median 65y.<br><br>**Sex**<br><br>Roux et al. (2013): 82% female<br><br>Majumdar et al. (2008): "when the results	Body functions and structures, activities and participation, personal factors and environmental factors	The review findings suggest that a person-centered and integrated care model can effectively address fragility fractures in older individuals by utilizing digital health technologies. This approach can lead to positive outcomes. It emphasizes the importance of incorporating resources for pain management, physical activity, nutrition, sleep hygiene, and mental health into the care model. The mere provision of health information is not enough; true education and understanding are essential. The review also underscores the significance of monitoring progress and providing feedback, as well as incorporating techniques like behavior change and motivational interviewing. It highlights the necessity of co-designing or co-creating solutions within specific practice contexts, considering available resources, to ensure a feasible and practical care pathway.	Not reported	**Cost**<br><br>"Further, patient-side costs and community feedback mechanisms also need to be considered." (Authors' comments)<br><br>**Access**<br><br>"Looking at the post-discharge care pathway; patients may need to attend orthopaedic out-patient departments (OPD), located in hospitals where access can be difficult, as they rely on family or ambulance services to provide transport. In addition, for falls prevention, patients may need to access specialist geriatric services and similarly, general practitioners (GP) within community for management of any existing co morbidities and osteoporosis."

	were stratified by sex, men in the interventio n group were less likely than women to receive appropriate care as15% and 44% respectivel y.”				
**Yeroushalmi 2020 Literature review N=28**	MS<br><br>**Age and sex**<br>Not provided	Body functions and structures, activities and participation, personal factors	This review presents evidence supporting telemedicine as an affordable and accessible platform for clinical care for people with pwMS, particularly for remote clinical examinations, longitudinal management, and telerehabilitation. However, the reviewed telemedicine studies were relatively small, highlighting the need for further research with larger sample sizes and greater methodological consistency. Additionally, there is limited data on the utilization patterns of telemedicine by pwMS. To optimize telemedicine services, it is crucial to engage in discussions with patients, healthcare providers, insurance companies, and policymakers. Further investigation into these issues can contribute to the development of effective telemedicine strategies	Not reported	**Cost**<br><br>Of the 28 studies meeting our criteria for the study, 18 were found to be low-cost interventions and 10 were considered medium cost for pwMS using our metric. Low-cost modalities typically required internet/phone access and hardware such as a webcam for CVT. Telephone hotlines and web-portal systems proved to be cost effective methods of long-term management and remote assessments (disability, cognitive, etc.). Telerehabilitation studies which relied on in-home exercises and did not utilize extra hardware or equipment were low cost as well.”

			that benefit pwMS, their families, and healthcare providers.		**Access**<br>“Web-based systems and telephone hotlines were shown to provide patient access to their providers during non-clinic hours from the convenience of their home.”<br><br>*Rural access*<br>“Finally, healthcare provider education and case consultation was useful in extending MS care into rural regions of the Pacific Northwest.”
**Zhou 2019 SR N=11**	People with disabilities (MI, DE, MS, TBI, MSD, Stroke, fracture, CI, JI, and facio scapulo-humeral muscular dystrophy)<br><br>**Age**<br><br>0 to 83y<br>Average: 60y<br><br>**Sex**	Body functions and structures, activities and participation, personal factors and environmental factors	Only a limited number of small-scale studies have explored digital interventions for individuals with disabilities in underserved areas. While these studies generally reported positive outcomes, their findings are insufficient to establish the effectiveness of telehealth-based digital interventions for enhancing the well-being of people with disabilities. This limitation arises from the small sample sizes and the absence of randomized controlled trials in the research.	**Autonomy**<br><br>Clark et al, (2002) reported in their outcomes: “Patient was functionally independent in household walking and self-care.”	**Cost/access**<br><br>“The benefits of telehealth include access to high-quality care, reduced travel time and costs, and increased collaboration among health care providers.”<br><br>“In recent years, as technologies have become ubiquitous and costs have declined, it has become easier to support the use of telehealth.”<br><br>Clark et al, (2002) study reported cost and travel savings outcomes.<br><br>Barlow et al, (2009) study reported “travel cost

	"Overall gender distribution in these11 studies was balanced. The gender of the children in these studies was not reported."				savings; rural therapists spent more time in preparation and follow-up; and clients had shorter wait times for assessment than rural face to face clients."<br><br>Kelso et al, (2009) study reported "cost savings for delivering early interventions via telehealth."<br><br>Schein et al, (2010) study reported "saved money and time."<br><br>Olsen et al, (2012) study reported cost savings and increased availability of services from specialists… "79% were satisfied with the telehealth experience; and telehealth removed time and travel barriers and increased availability of qualified personnel."<br><br>Levy et al, (2015) study reported "96% of patients were satisfied with the telehealth experience; and avoided travel miles, driving time, and travel reimbursement."<br><br>"Overall, these care providers were generally satisfied with this digital intervention delivery approach as it provided

					intervention results comparable with face-to-face visits, increased patient contact time, and reduced travel time and costs."<br><br>*Rural vs. urban*<br><br>Ten of the included studies were for people with disabilities in rural areas and one was for people in urban area.<br><br>*Internet access*<br><br>"The wide adoption of the internet and smart mobile devices in recent years indicate that the availability of technology is not an issue anymore."<br><br>**Ethnicity**<br><br>"Most of the studies also did not report the race of the participants. The ones that did indicated that most of the participants were white."
**Hao (2023) SR and Meta analysis N=18**	Cerebral palsy<br><br>**Age**<br>Ave. 10,1<br><br>**Gender**	"Body functions and structures, Activity, and Participation outcomes based on the International Classification of Functioning, Disability and	"Overall, virtual reality appears feasible and effective in home-based rehabilitation programs for children and adolescents with cerebral palsy. Home-based virtual reality may serve as an adjunct to conventional facility-based physical therapy to promote participation in therapeutic exercises and maximize	**Adverse events**<br><br>- In three cases, one participant in the experimental group had seizures which was not related to virtual reality.<br>- 11 reported muscle soreness, 10 reported	**Cost**<br><br>"Home-based rehabilitation for cerebral palsy has been proven to be a cost-effective service model (Azar et al., 2015). It enables children and families to participate in therapeutic activities built into their daily routines

	Usually not specified, but, one with three girls with cerebral palsy	Health (ICF) framework"	rehabilitation outcomes. Effects of home-based virtual reality on the three ICF domains were revealed including (1) Body function and structure (i.e. upper extremity function, gross motor function, strength, bone density, and cognition); (2) Activity (i.e. balance, walking, and daily activity performance), and (3) Participation. Meta-analyses revealed significant improvements in hand function, gross motor function, and walking ability following home-based virtual reality intervention."	fatigue, 8 reported falls; no serious injury reported.<br><br>**Autonomy**<br><br>"Rehabilitation is essential for children and adolescents with cerebral palsy to improve mobility and self-care independence, which promote quality of life and social engagement (Aisen et al., 2011)."(Introduction)<br><br>"Results suggested that integrating virtual reality technology to deliver and enrich home-based rehabilitation for children and adolescents with cerebral palsy is feasible. Effects of this approach were revealed and qualitatively synthesized according to ICF domains including Body function and structure (i.e. upper extremity function, gross motor function, strength, bone density, and cognition); Activity (i.e. balance, walking, and daily activity performance); and Participation." (Discussion)	without the barriers of arrangement and transportation to facilities." (Introduction)<br><br>"The long-term disability related to cerebral palsy imposes challenges to the lives of children and their families and creates extensive economic burdens on society (Tonmukayakul et al., 2018)." (Introduction)<br><br>"Another consideration for the application of home-based virtual reality is the cost. Only one included study analyzed the economic aspects of program implementation. Farr et al. (2021) concluded that using Nintendo Wii Fit at home was inexpensive, with £20 per child. However, this calculation was based solely on the expense of therapy time and not the cost of the instrument. Three included randomized controlled trials (James, Ziviani, Ware, and Boyd, 2015; Mitchell, Ziviani, and Boyd, 2016; Piovesana et al., 2017) used the Mitii system, a web-based multimodal therapy developed for neurorehabilitation. The cost-effectiveness analysis was conducted in another

					study (Comans et al., 2017) finding the cost per responder of this system ranging from AU$3078 to AU$4191 depending on the outcome measured. While a scoping review (Aliprandi, Pan, Mosley, and Gough, 2022) suggested virtual reality may be a cost-effective adjunct to outpatient homebased programs, further formal health economics analyses with a comprehensive consideration of cost variables associated with the clinical application of homebased virtual reality on a large scale are warranted" (Discussion / Home-Based Virtual Reality Device Selection)<br><br>**Access**<br><br>"However, due to limited health-care resources, there is an unmet need for them to access rehabilitation services continuously (Colver, Fairhurst, and Pharoah, 2014)" (Introduction)<br><br>**Age**<br><br>"However, although previous systematic reviews examined home-based virtual reality in adults with

					neurologic conditions (i.e. Parkinson's disease) (Gallou-Guyot et al., 2022) and multiple sclerosis (Dalmazane et al., 2021), there is a lack of a systematic evaluation of its application in the pediatric population, especially for children with cerebral palsy." (Introduction)
**Thwaites (2023)**<br>**SR**<br>**N=17**	Falls related outcomes in community dwelling adults with neurological conditions: stroke, MS, Parkinson<br><br>**Age**<br>Ave. = 59,7 (from 34,46 to 70 years)<br><br>**Gender**<br><br>**Males** : ave. 49,25% (from 20% to 74%)	Body functions: - Original data involving falls risk or rates, standing balance, fear of falling, environmental safety or falls prevention knowledge	**Adverse events**<br><br>"Adverse event reporting was limited, and no study compared adverse events between telehealth and comparator interventions."<br><br>"There was low-quality evidence that telehealth interventions improve balance outcomes more than face-to-face interventions (pooled between-group mean difference 2.48 Berg Balance Scale units, 95%CI 0.77 to 4.20). Fear of falling was not different between intervention delivery modes."<br><br>"This review did not find evidence to support or refute the greater effectiveness of telehealth interventions in reducing falls risk or rates compared to in-person delivery. The findings from the one trial in our review that included falls prevention data	**Autonomy**<br><br>"In contrast to our findings, a review of telehealth interventions for older adults found a small significant improvement in falls self-efficacy, and suggested exercise combined with motivational support improves balance confidence."	**Access / Costs**<br><br>"Telehealth delivered via synchronous videoconferencing may help to address known barriers to accessing falls prevention interventions including transport, distance, costs and fatigue." (Introduction)<br><br>"Indeed, only one study 55 in our review provided details regarding costs to deliver telehealth interventions, highlighting that more information is needed." (Discussion)

			should be interpreted cautiously due to the short follow-up time frame and risk of bias.51 The lack of evidence on falls is perhaps unsurprising given that large sample sizes and long follow-up periods are needed to robustly evaluate effectiveness for falls reduction. However, the findings across the included studies suggests that synchronous telehealth interventions are safe, feasible and can lead to at least comparable improvements in falls-related outcomes" (Discussion)		

Note: pwMS=Multiple Sclerosis, TBI=Traumatic Brain Injury; MS=Multiple sclerosis; SCI=. Spinal Cord Injury; TKA= Total Knee arthroplasty; pwMS=People with multiple sclerosis; FDA: The United States Food and Drug Administration; N= Number of studies included; MI= mobile impairment; DD=developmental disability; MSD=Musculo-skeletal disorder; CI= Cognitive impairment; JI= Joint replacement, TKA= Total knee arthroplasty and ICT=internet and communication technologies.
