# Supplementary material for: A Rapid Review of Ethical and Equity Dimensions in Telerehabilitation for Physiotherapy and Occupational Therapy
Source: Int J Environ Res Public Health. 2025 Jul 9;22(7):1091. doi: 10.3390/ijerph22071091 (PMC12294586; doi:10.3390/ijerph22071091)
Supplement: Supplementary file 1 [file ijerph-22-01091-s001.zip › Table S2- Physiotherapy study characteristics (N=42) Apr 02 2025.pdf]

Table S2: Physiotherapy study characteristics (N=42)

Study#	Study Author<br>Year<br>Study Design<br>N# included studies	Participants<br>Age<br>Sex<br>Morbidity<br>/Population	Key findings	Ethical principles	Equity aspects
**1**	Almojaibel<br>(2016)<br>NRS<br>N=17	COPD<br>**Age and Gender:** Not reported	The narrative review highlights the feasibility and acceptability of telehealth for real-time interactive pulmonary rehabilitation services for COPD patients at home. Cost-effective telerehabilitation systems, utilizing readily available equipment and free videoconferencing software, are safe and enhance access to pulmonary rehabilitation, particularly benefiting patients in rural locations. Importantly, the participants' computer literacy level does not impact the acceptance or utilization of tele-pulmonary rehabilitation systems.	**Adverse events**<br><br>Two included studies reported adverse events and both concluded that the utilization of telerehabilitation was deemed safe, with no occurrences of major or moderate adverse events.<br><br>**Autonomy**<br><br>"Self-monitored, home-based pulmonary rehabilitation is an alternative<br>method that can be more convenient and accessible to deliver pulmonary rehabilitation services compared to in-patient programs.<br>Self-management outcome included." (authors' comment in the introduction)	**Cost**<br><br>"Low-cost models of telerehabilitation systems, using available equipment and free videoconferencing software programs, are safe to use and can improve access to pulmonary rehabilitation services, especially for patients living in rural areas." (authors' comments in the conclusion)<br><br>**Access**<br><br>In one of the included studies, Tabak et al. 2014) reported that "recruiting patients was difficult because of the strict inclusion and exclusion criteria that included the need for a computer with Internet access at home, which was not always possible for elderly patients."<br><br>**Digital literacy**

					"It is worth noting that participants' computer literacy level has no effect on acceptability or on the utilization of the tele-pulmonary rehabilitation systems." (authors' comments in the conclusion)
**2**	Appleby (2019)<br>SR<br>N=13	Stroke<br><br>**Age and Gender**<br>Age ranges from 28–85 years and a gender bias towards men (more male participants than women).	Telerehabilitation is gaining popularity for providing equitable access to care, with evidence suggesting its effectiveness in motor function, ADLs, independence, and quality of life. However, implementing telerehabilitation requires dedicated resources, infrastructure, and specialized training for health professionals. Patient perspectives and preferences, along with uncertainties about its perceived inferiority to traditional care, must be considered. Additionally, due to literature gaps, no specific recommendations regarding ideal timing and parameters for telerehabilitation are currently available.	**Adverse events**<br><br>"No adverse effects reported" (authors' comments)<br>**Autonomy**<br>Five studies reported Independence and self-efficacy outcomes.<br>"The summarised findings from this review suggest that telerehabilitation may be as effective as usual care for motor function, ADLs, independence and satisfaction/ quality of life."	**Cost**<br><br>One study, conducted by Piron et al. in 2009, presented cost outcomes in dollars.<br><br>In conclusion of the SR, the authors highlight crucial knowledge gaps at the practical level, emphasizing the need for ongoing research on aspects like the training requirements for health professionals, necessary infrastructure, ongoing maintenance costs, and patient preferences.
**3**	Batsis (2019)<br>SR<br>N=17	Older adults with chronic disease entities, including	Telemedicine is both feasible and acceptable for delivering care to older adults. Future research	**Autonomy**<br><br>One included study Treif (2013) added self-care	**Cost**<br><br>"While a number of observational studies and

		neurological disorders, depression, chronic obstructive pulmonary disease, diabetes, or high-risk older adults with different baseline characteristics)<br><br>**Age and Gender**<br><br>The average age of the participants was 65 years or older, with none being younger than 60 years. The mean age ranged from 65.1 years to 86.45 years. F= 1768 and M= 1765	should prioritize well-designed randomized trials to address biases observed in current syntheses. Clinicians are encouraged to incorporate telemedicine into routine practice to overcome distance and access barriers to care.	Activities scale as primary outcome.	single-site pilot studies suggest that TMed may have long-term cost-effectiveness, may reduce hospital utilization or emergency department visits, data in ambulatory settings have been less commonly evaluated.” (authors’ comment in the introduction)<br><br>**Access**<br><br>“In the study conducted by Vahia (2015), the objective was to assess the comparability of neuro-cognitive assessments through telepsychiatry and in-person methods for older rural Latinos.”<br><br>“Few studies, though, focused specifically on rural adults and the results were mixed. While telemedicine may provide a unique opportunity to reach isolated, low-resource populations with limited access to in-person medical services, well-designed, high-quality studies are needed.” (author’s comment in the discussion)<br><br>“TMed may help provide effective care, particularly in rural and underserved areas,

					and executing the Institute of Medicine's recommendation to advance TMed resources is strongly supported by our observations." (author's comment in the discussion)<br><br>**Digital Divide**<br>"Few studies described technological issues, particularly in areas with poor bandwidth, likely due to the urban-rural divide observed." (author's comment in the discussion)<br><br>"While a number of RCTs using TMed in non-hospital settings exist, well-designed, powered trials will provide guidance in using this technology in older adults, particularly in rural areas." (author's comment in the discussion)<br><br>**Socioeconomic status**<br><br>"Socioeconomic status was indicated in nine studies, and patient frailty or functional status was inconsistently reported using different indices."<br><br>"The majority of studies did not highlight functional or socioeconomic status suggesting a need for future studies to report on these

					parameters." (authors' comment in the discussion)
**4**	Berton (2020)<br>SR<br>N=24	Orthopedic patients (OR, fracture, RA and chronic non-specific low back pain, syndrome and scapular dyskinesia found in the hip, knee and ankle joint)<br><br>**Age**<br>The majority of studies (79%) focused on patients aged 40 to 60 years, with 12% involving participants aged 60 to 80 years. A smaller proportion, 9%, examined patients between 25 and 30 years of age.<br><br>**Gender** not reported.	This review assessed remote virtual technologies for orthopedic rehabilitation, identifying nine RCT and 15 non-randomized studies. Despite the limited quantity, the studies showed high or moderate quality with low bias risk. Heterogeneity among studies excluded meta-analysis. Lack of standard procedures, diverse equipment, and outcome measurement methods were noted. Age and social context affected adaptability to technology, influencing treatment compliance and outcomes. A strong patient-physiotherapist relationship is crucial, and remote technologies facilitate clinical interactions. These technologies offer cost-effective high-quality care, addressing the rising demand for orthopedic rehabilitation. Future research should develop objective methods to 5evaluate the clinical quality of new technologies and	**Adverse events**<br><br>"The risk of this technology is the impossibility to recognize real dangers that can cause injuries. In AR, virtual reality and real reality overlap and the patient is aware of potential dangers. (authors' comment in the "introduction)<br><br>Real-time monitoring of patients' physiotherapy poses a challenge, but analyzing user activity as it occurs can help prevent training pitfalls. This challenge can be addressed through programmed visual-optic feedbacks tailored for specific tasks. (authors' comment in the discussion)<br><br>**Autonomy**<br><br>One of the include studies Naeemabadi (2020) reported "A higher level of self-confidence was reported" as outcome." In the same study "Patients reported an increase in self-esteem themselves and an improved relationship with the	**Costs**<br><br>"It is recommended that these technologies should be further improved and their fields of application should be expanded as they allow the delivery of high-quality care at reduced costs."<br><br>"Orthopedic remote rehabilitation leads to reduced costs for the national health system. As already proven in other medical fields, decreased costs are related to the reduction of transports, hospitalizations, and readmissions. It is assumed that for patients living more than 30 km away from the rehabilitation center, savings are around 230 dollars."<br><br>"Moreover, remote virtual rehabilitation allows continuous monitoring of several patients at the same time, saving time and money. The possibility to deliver high-quality care at reduced costs is necessary given the growing demand for orthopedic rehabilitation and increasing costs related

			conclusively demonstrate the advantages of VR, AR, gamification, and telerehabilitation over face-to-face orthopedic rehabilitation.	physiotherapist despite the distance."<br><br>**Handling data/data protection/data security**<br><br>"Future developments should focus on adequate data storage systems and real-time analysis of continuous updated information, to provide immediate feedback to patients. Those systems should also guarantee privacy protection." (authors' comment in the discussion)	to it." (authors' comment in the discussion)<br><br>**Access**<br><br>"This system is advantageous not only for those who live far from rehabilitation centers but also for people with severe disabilities as moving is not necessary." (authors' comment in the discussion)<br><br>**Digital Literacy**<br><br>In the Babic (2019) study, patients initially struggled with adapting to new technology, but the majority reported experiencing physical improvements. In the Nelson (2017) study, only 35% of participants expressed confidence in using technology.<br><br>"Age and social context influence adaptability to technology. For elderly patients, it is challenging to approach technology, while younger patients are predisposed to it. Therefore, compliance to treatment and outcomes can be affected by the patient's perception of technology. As the majority

					of orthopedic patients are of medium-high age, the user-friendliness of remote virtual technology should be guaranteed. More and more simple platforms have been created. They do not require complicated software or installation of multidirectional cameras. It is merely necessary that the patient has a computer or a smartphone to connect to the Internet." (authors' comment in the discussion)<br><br>**Education and Employment status**<br><br>Forty-three percent of participants held a university degree, with the majority falling in the 40–60 age group. In this age group, 27% were workers, 8% were unemployed, and 0.5% were retired. The 60–80 age group constituted 12% of the study, with 10% being retired, 2% workers, and 0.5% semi-retired. The third age group, 25–30 years, made up 9%, and all participants in this group had a university degree.
**5**	Blood (2019)<br>Narrative review<br>N=12	Cardiac surgery, MS, SCI, Stroke	"Additional randomized-control trials are recommended to establish if telehealth practices	**Adverse events**<br><br>"Patients that have more complex medical diagnoses	**Cost and access**<br><br>"In addition, there is currently no uniformity

		**Age and Gender**<br>**NR**	result in equivalent outcomes to standard physical services Continuing education opportunities should be designed for clinicians to become more knowledgeable on appropriately used telerehabilitation, current state and federal guidelines, and billing procedures. Physical therapy student education programs need to incorporate these methods as well to allow new graduates to have solid knowledge base on these methods. As technology continues to advance, physical therapists in all settings need to say up-to-date with the latest trends and continue to find new ways to serve all patients."	or multiple co-morbidities may choose not to participate in telerehabilitation from fear of ineffective management of complications that could arise during a treatment session."<br><br>**Handling data/data protection/data security**<br><br>"The provision of services via telehealth delivery requires both start-up costs for devices and software, as well as fees for maintenance and security."	between states with regards to practice acts and the ability to provide physical therapy remotely. Reimbursement for services may also be a barrier; Medicare is not currently reimbursing for physical therapy services delivered remotely."<br><br>"The use of health and rehabilitation related applications on smartphones may have widespread benefits to certain populations but may not be available to all in need due to the high cost of the devices even when patients are granted rebates to purchase them."<br><br>"Research has demonstrated that this method of service delivery is considered acceptable by both clinicians and patients and can have a positive impact on the ability to keep cost of care down."<br><br>**Digital Divide (Internet Access)**<br><br>"Patients who are willing to participate in videoconferencing therapy sessions must also have a reliable and high-speed internet connection, which

					may limit the areas where this technology can be used."<br><br>**Digital Literacy**<br><br>"In addition, in order to have a visual connection with the therapist, the patient must feel confident with a computer and software that al-lows for videoconferencing; this may be perceived as difficult to use."<br><br>"Those that are involved in physical therapy education must incorporate teaching telerehabilitation methods, as well as on the state and federal guidelines for such services, to ensure that the next generation of physical therapists are knowledgeable and able to provide for them appropriately. Physical therapists working in all settings must stay current with regulations and pursue continuing education opportunities to allow them to better serve all patients, especially those that experience the barriers discussed that make them the most vulnerable to inad-equate access to care."

**6**	Chan (2016)<br>SR and MA<br>N=9	Cardiac and Pulmonary Rehabilitation<br><br>**Age and Gender**<br><br>Age Mean between 50 to 65.7<br><br>More than 55% of the included studies involved male participants.	This review of nine studies suggests that TR interventions, monitored by healthcare practitioners, provide a viable and effective option for improving access to rehabilitation for CVD and COPD patients. The benefits seen in TR interventions appear comparable to standard exercise components in CR and PR programs. Physiotherapists considering TR interventions can expect similar outcomes if technology and monitoring align with those in the reviewed studies. The findings emphasize the need for further research to explore barriers and factors affecting the implementation of TR interventions in CR and PR, advocating for both quantitative and qualitative approaches to address challenges in program implementation and participant motivation.	**Adverse events**<br><br>Adverse events were examined in seven of the eight CR studies and the PR study with no reported adverse events during any exercise sessions. No adverse events reported in the included studies (author's comment).	**Cost**<br>NR<br><br>**Access**<br><br>"TR programmes with exercise components that include telemonitoring may improve exercise-related outcomes, better ensure patient safety during exercise, and ultimately improve access to rehabilitation interventions." (authors' comment in the introduction).<br><br>One study reported "rural patients travelled to a setting closer to them where on-site junior exercise physiologist monitored pulse oximetry, blood pressure, and RPE via portable telemetry." Dalleck (2010)
**7**	Chen (2020)<br>SR and MA<br>N=21	PD<br><br>**Age and Gender**<br>Mean age: 58.4 to 80.63	"Telehealth intervention is an effective option for individuals with PD to improve their motor impairment. Further well-designed studies are	**NR**	**Cost**<br>"In addition, telehealth helps to reduce travel time, and is associated with lower overall health costs."

		**Gender:** NR	warranted to confirm our findings.”		(authors’ comment in the discussion)<br><br>**Access**<br><br>“In spite of these adverse symptoms, the literature indicates that a great number of PD patients have limited access to conventional face-to-face healthcare due to factors such as a distance barrier, financial burden, mobility difficulties, or lack of time.” (authors’comment in the introduction)<br><br>“As the population ages and people’s requirements for health care grow, increasing access to health care has become an important issue worldwide. However, clinic-based programmes are inaccessible for many patients due to distance, disability, and the distribution of physicians.”<br><br>“Another, larger, challenge concerns the inconsistency and insufficiency of financial reimbursement and insurance coverage.”<br><br>“Some countries, including Germany and Denmark, provide reimbursement for tele-medicine services.22 These countries always have

					a single government payer or a centralized medical records system. However, elsewhere this is not the case: in the United States for example, telehealth is reimbursed by Medicare health insurance in designated Centers within rural areas, but is not in many larger communities, despite their population size." (authors'comment in the discussion)
**8**	Chirra (2019)<br>NRS<br>N=102	Neurological diseases, (Stroke, MS, epilepsy, PD, pediatric neuro oncologic, chronic disease and depression, CVD)<br><br>**Age and Gender**<br>NR	Integrating telemedicine and telemetry into clinical practice is a significant challenge in modern medicine. The potential benefits include managing complications of chronic conditions through a home-based, patient-centered care model. However, achieving this requires collaborative efforts from various stakeholders to develop an interoperable software platform. The goal is to provide a holistic and accessible approach to care, addressing disparities and ensuring an easy-to-use, secure, and cost-effective ecosystem. The review acknowledges its narrative structure as a limitation and suggests the need for further systematic	**Adverse event**<br><br>"Telemetry also has applications in patients requiring in-tensive care, such as in the case of neoplastic diseases, where in pharmacological treatments require continuous monitoring of adverse events (e.g., vomiting, dehydration, and so on) and vital signs (e.g., blood pressure, blood glucose levels, pulse rate, and so on)."<br><br>**Handling data/data protection/data security**<br><br>"Critical challenges include the integration of the systems for data monitoring with an easy-to-use, secure, and cost-effective platform that is both widely adopted	**Cost/access and digital Divide**<br><br>"Another challenge for the widespread diffusion of tele-medicine is the cost of technologies, and the difficulty of integrating them into challenging environments such as in the home of elderly patients, communities with low educational status, low-middle income countries, and rural areas with incomplete or absent technology penetration."

			studies to better understand telemedicine's application to neurology.	by patients and healthcare systems and embraced by international scientific societies.”	
**9**	Cottrell (2017)<br>SR and MA<br>N=13	Musculoskeletal conditions<br><br>**Age**<br>Age ranged from 37.6 to 75 years.<br>**Gender:** NR	“Real-time telerehabilitation appears to be effective and comparable to conventional methods of healthcare delivery for the improvement of physical function and pain in a variety of musculoskeletal conditions.”	**Autonomy**<br>Self-efficacy was the primary outcome measure in only one trial. Pariser, D (2005)<br><br>“Self-efficacy has been defined as “the degree of confidence an individual has in carrying out a specific activity”. Interventions specifically targeting self-efficacy are considered to be critical in ensuring compliance to management strategies, subsequently favourably influencing health outcomes of a chronic musculoskeletal pain population. Whilst unable to provide any formal conclusions with respects to telerehabilitation and its effect on self-efficacy, this area does warrant further robust clinical trials considering the direct impact chronic physical diseases has on the likelihood of developing comorbid psychological disorders.” (authors’ comment in the discussion)	**Cost**<br><br>“Whilst such initiatives have reduced the cost and wait time for orthopaedic health services, poor access, as a result of geographical isolation or local service availability, continues to restrict appropriate and timely care for many individuals.”<br><br>**Access**<br><br>“Whilst such initiatives have reduced the cost and wait time for orthopaedic health services,5–7 poor access, as a result of geographical isolation or local service availability, continues to restrict appropriate and timely care for many individuals. Lack of access to appropriate musculoskeletal care may be potentially overcome by the use of real-time telerehabilitation.” (authors’ comment in the introduction)<br><br>“This review does have important clinical

					implications. Whilst the majority of musculoskeletal conditions can be effectively managed with the input of either medical or allied health professionals, access to appropriate healthcare services are limited in rural and remote communities, which houses a population that is 2.5 times more likely to suffer from an arthritic condition than their urban counterparts. A lack of local resources forces individuals to travel significant distances, providing even further economic burden on both the individual and society."<br><br>"Telerehabilitation is another avenue to providing equitable healthcare delivery following these surgical procedures for those individuals that are unable to access conventional face-to-face care within their local district**.** (authors' comment in the discussion)
**10**	Davis (2012)<br>NRS<br>N=8	Osteoarthritis<br><br>**Age and Gender**<br>NR	"Three studies highlighted differences between observed performance measures and self-reported difficulties in physical function, emphasizing limited evidence for the effectiveness of manual therapy in hip and knee		**Cost and Access**<br><br>"Access to rehabilitation professionals is limited for a variety of reasons including too few practicing professionals per population, cost of service and geographic factors where

			osteoarthritis. Two studies noted varying recovery times after total knee replacement. Evaluation of participation measures revealed distinctions between activity limitations and participation. Tele-rehabilitation emerged as a promising option, particularly in rural areas post-joint replacement. The importance of incorporating both performance measures and patient-reported outcomes in osteoarthritis assessments was emphasized, with a call to consider participation measures as core outcomes.”		individuals living in rural or remote communities must travel long distances for care. Hence, alternative methods of care delivery using technology are attractive and may have potential to increase and ease access to care. Telemedicine has generally been used to promote access to specialist care and has been shown to be feasible and acceptable to patients.”
**11**	Du (2020)<br>MA<br>N=8	Chronic Low back pain<br><br>**Age**<br>≥ 18 years older<br><br>**Gender:** NR	“This research found moderate to low evidence that e-Health based self-management programs played a positive role in im-proving pain intensity and disability within short-term period for chronic low back pain patients. However, it remains un-clear whether the effects can be sustained. More rigorously de-signed randomized controlled trials are warranted to determine the optimal delivery mode and	**Autonomy**<br><br>“The effectiveness of e-Health self-management programs for chronic low back pain varies based on evidence quality. Moderate-quality evidence supports a clinically important pain relief effect at immediate and short-term follow-ups, but very low-quality evidence shows no significant effect at intermediate follow-up. For disability, there's a clinically important improvement at	**Cost**<br><br>“Many factors, including distance, time and cost, have caused numerous barriers to reach skills and resources of self-management for ordinary citizens. It requires alternative modality to address the problem (Martorella et al., 2017).” (authors’ comment in the introduction)<br><br>**Access**<br>“More importantly, much more people have access to

			duration of e-Health based self-management programs for chronic low back pain, and long-term follow-up is also required.	immediate follow-up with moderate-quality evidence, but low to very low-quality evidence indicates no significant difference at short-term and intermediate follow-ups, with a favorable trend at short-term follow-up.”<br><br>“Numerous studies have confirmed the positive effects of self-management model for non-communicable chronic diseases and cancers. We also performed a systematic review and meta-analysis to investigate its effect on chronic low back pain, with a conclusion that self-management programs had a moderate effect in relieving pain, and small to moderate effect in improving disability However, many factors, including distance, time and cost, have caused numerous barriers to reach skills and resources of self-management for ordinary citizens. It requires alternative modality to address the problem.” (author’s comment in the introduction)	smart phone than to desktop personal computer, especially in developing and countries such as China and India (Digitimes).
**12**	Flodgren (2015)<br>SR<br>N=93	Cardiovascular disease (36), diabetes (21), respiratory	This review suggests that telemedicine (TM) in heart failure management yields comparable health	**Autonomy**	**Cost**

		conditions (9), mental health or substance abuse conditions (7), conditions requiring a specialist consultation (6), co morbidities (3), urogenital conditions (3), neurological injuries and conditions (2), gastrointestinal conditions (2), neonatal conditions requiring specialist care (2), solid organ transplantation (1), and cancer (1)<br><br>**Age**<br><br>The mean age in the intervention group ranges from approximately 38.6 to 77.1 years.<br>The mean age in the control group ranges from	outcomes to traditional face-to-face or telephone care delivery. Evidence supports TM's capacity to enhance blood glucose control in individuals with diabetes. However, the overall cost of health service and the acceptance levels among patients and healthcare professionals remain uncertain due to limited available data. The effectiveness of TM is likely influenced by various factors, including characteristics of the study population (such as the severity of the condition and disease trajectory), the specific function of the intervention (whether for chronic condition monitoring or access to diagnostic services), and the nuances of the healthcare provider and system implementing the intervention.	Education, advice for self-management, and support was reported in 23 studies<br><br>**Adverse event**<br><br>"Technical difficulties, for example failure of data transmission and/ or video-conferencing, were reported in only six studies. One study (Nguyen 2008; N = 50) reported that technical difficulties led to a high drop-out rate (43%) in the intervention group; a second study (Schwarz 2008), reported that 20% of intervention patients were unable to begin transmission of data and another study. (Chiantera 2005), reported that 10% of patients in the TM group left the study as they could not use the TM device. One study (Crossley 2011), reported failure of the TM monitoring system to send an automatic clinician alert in 246 of 575 cases (42.8%); this was because the alert was programmed to 'off' (7%) or was not	"Two studies (Benatar 2003; N = 216; Giordano 2009; N = 460) of remote monitoring with automated alerts or risk stratification reported lower hospital re-admission costs for TM as compared with usual care; three studies (Dar 2009; N = 182; Dendale 2012; N = 160; Schwarz 2008; N = 102) reported no difference in total health service costs between groups. One study of TM with video-conference (Jerant 2001, N = 25) reported lower hospital re-admission costs for TM as compared with usual care . Two studies (Al Khatib 2009; Crossley 2011) reported no difference between TM and usual care in hospital admissions, ED visits or unscheduled visits at 12 and 15 months follow-up. Two studies reported shorter LOS in TM as compared with control, (Crossley 2011; Halimi 2008). One study (Al Khatib 2009), reported higher mean total healthcare cost per TM patient as compared with usual care and two studies lower costs for TM (Crossley

		approximately 41.4 to 79.1 years.<br><br>**Gender**<br>Intervention Group:<br>Range of male percentages: 27.1% to 100%. Some studies provided numbers or indicate no information.<br><br>Control Group:<br>Range of male percentages: 14% to 100%. Some studies provided numbers or indicate no information.<br><br>Some entries lack specific information or state that age data is not reported.<br>The age data vary across studies, with some showing similar mean ages in intervention and control groups,		reset after being previously triggered (93%). In addition, when an automatic clinician alert was triggered it resulted in a successful transmission in 180 (55%) cases, while for 149 (45%), clinical events automatic clinician alerts were triggered but not successfully transmitted. One study (Wong 2006), reported a 30% failure rate for video-consultations, which was mostly due to technical errors and logistic difficulties at the referring institution. One small study (King 2009), recruiting people attending an addiction treatment service, reported that 30% of participants experienced computer and adherence related problems."	2011; Halimi. One study (Parati 2009) reported no differences between groups for costs of examinations and overall cost of patient management at six months. One study (Biermann 2002, data from Biermann 2000, N = 48) reported lower healthcare costs per year for TM patients as compared with usual care patients, and one study (Jansa 2006, N = 40) lower costs for TM when delivered without technical problems. Chase 2003 reported lower costs for TM as compared with face-to-face clinic visits. In one study (Boaz 2009, N = 35), costs were increased in the usual care group due to more unscheduled visits.<br>A cost analysis in one study (Palmas 2010, an additional report from Shea 2006), reported slightly higher mean annual Medicare payments in the TM compared with the usual care group. One study (Crow 2009), an additional report from Mitchell 2008, N= 128), reported lower total costs per abstinent participant for TM

		while others exhibit differences.			as compared with usual care, with the difference mostly pertaining to the travel costs of the therapist; a second study (Ruskin 2004, N = 119), reported higher costs for TM, but no differences between groups when the therapists' travel costs had been accounted for. Two studies (Hopp 2006, N = 37; Noel 2004, N = 104), reported no difference in healthcare resource utilisation between groups. One study (Finkelstein 2006), reported more patients receiving usual care being transferred to a higher level of care at six months as compared with TM group patients. This study also reported lower cost per visit in the video- and monitoring group. Noel 2004 (N = 104), which compared costs six months before the intervention and costs during the six-month intervention, reported a greater decrease in the average healthcare costs per participant in the TM group.”

					**Ethnicity**<br><br>The racial and ethnic distribution in different intervention and control groups indicates a range of percentages and specific instances across various studies or contexts.<br>54 studies did not report ethnicity.<br><br><br>Caucasian/White:<br>Intervention: Ranges from 62% to 92%<br>Control: Ranges from 64% to 100%<br><br>African American:<br>Intervention: Ranges from 0% to 29%<br>Control: Ranges from 0% to 46%<br><br>Hispanic or Mexican Origin:<br>Intervention: 100% in one study<br>Control: Information not provided in the summary<br><br>South Asian:<br>Intervention: 20%<br>Control: 21%<br><br>Non-Hispanic White:<br>Intervention: 89.7%<br>Control: 73.0%<br><br>No Whites:

					Intervention: Instances with no information<br>Control: Instances with no information<br><br>White/Black Distribution:<br>Intervention: 57.5%/41.9%<br>Control: 47.7%/52.3%
**13**	Mani (2017)<br>SR<br>N=11	Musculoskeletal disorders<br><br>**Age**<br>NR<br><br>**Gender**<br><br>60 males and 62 females were reported in 7 studies.	This systematic review found that TR based physiotherapy assessment is technically feasible for measuring various outcomes in MSDs. The assessment showed good concurrent validity for pain, swelling, ROM, muscle strength, balance, gait, and functional outcomes. Lumbar spine posture assessment exhibited a low level of concurrent validity, while assessments of SOTs, NDTs, and scar assessments demonstrated moderate concurrent validity. Inter-rater and intra-rater reliabilities were generally good to excellent for TR-based physiotherapy assessment in MSDs.	**Autonomy**<br>Four studies measured static muscle strength by applying patients' self-resistance and Patients were asked to perform modified self-administered SOTs under the guidance of the TR physiotherapist.<br>Self-palpation was also used in one study for pain assessment.<br><br>**Risk of technology**<br><br>"Low image resolution due to poor bandwidth has made it difficult for the TR physiotherapist to guide the patient to palpate the anatomical location of the source of pain and tenderness.<br>This barrier may be overcome by providing a body chart via the TR system ahead of time.In addition, the validity of SOTs and NDTs via the TR method might be improved	**Access**<br>"Subjects in the reviewed studies did not represent real populations who would include those unable to travel due to chronic disability or aging or those who lived in rural areas with insufficient access to rehabilitation services."<br><br>**Access**<br><br>"who lived in rural areas with insufficient access to rehabilitation services."<br><br>"The incidence of MSDs is noted to be higher in rural populations due to the lack of avail-able healthcare and rehabilitation services.TR may be a potential solution to deliver remote rehabilitation services using information and communication technology to address this rural healthcare disparity." (comment introduction)

				by guiding and training the patients or caregiver through real-time feedback, supplemented by high-quality video or a video weblink. Poor rapport during the TR session may have a negative influence on clinical reasoning in the diagnosis of MSDs via TR.”	
**14**	Gilbert (2018)<br>SR<br>N=4	Orthopaedics<br><br>**Age**<br>NR<br><br>**Gender**<br>22 males and 42 females	All studies agree that patients find virtual consultations (VC) acceptable, but clinician acceptability was not assessed. The research suggests VC is acceptable in specific clinical contexts, but further qualitative studies are needed.	**Autonomy**<br><br>“It was useful in the early stages post-surgery where pain and disability can be prevalent. It was described as facilitating the patient’s transition from being dependent and passive to becoming an active participant in their rehabilitation.”	**Cost**<br>“A common theme running throughout the four studies was that of convenience. It is clear that accessing healthcare from a distance reduces cost and saves time for the patient. It was acknowledged by patients that this may be more important to some patients who do not have access to transport. In the United Kingdom, patients are at the centre of the NHS and patient’s are allowed to choose their NHS healthcare provider through initiatives such as ‘choose and book’. VC may provide new opportunities for patients wanting to choose services outside of their local geographical area.”<br><br>**Disabilities/challenges**<br><br>“Patients reported that VC was preferable to telephone as it increased their

					confidence in the clinician's diagnosis, and could be useful for patients who have difficulties with verbal descriptions."
**15**	Govercin (2010)<br>NRS<br>N=41	Stroke<br><br>**Age**<br>The age range summarized for all pilot trials was 13–88 years and 19–79 for RCTs.<br><br>**Gender**<br>NR	"Although general recommendations cannot be given, VR and TR approaches are feasible for upper-limb rehabilitation post stroke. Immersive VR approaches were shown to be effective compared to control groups. While non immersive VR did not prove to be effective, evidence from noncontrolled trials revealed that non immersive VR may prove to be effective compared to standard care."	**NR**	**Cost**<br>"The advantages here were low costs and positive results with regard to feasibility, motivation, availability, and a smaller degree of immersive-ness. he main reason for limited outpatient rehabilitation is still the high costs of a frequent, ongoing recovery program for the upper limb. Large, immersive and costly devices have now changed to small, smart, and intelligent devices at a lower cost." (comments discussion)
**16**	Grona (2018)<br>SR<br>N=17	Musculoskeletal conditions<br><br>**Age**<br>age mean range from 24 years to 62 years<br><br>**Gender**<br>One study reported 3 females and 1 male	"Validity and reliability studies were identified as having high risk of bias. Intervention studies were of moderate quality, and found positive impact on health outcomes and satisfaction. Two studies evaluated costs, with evidence of cost savings in one study. More robust research is required to evaluate long-term effects of telerehabilitation for physical therapy management of	**NR**	**Cost**<br>"Two studies evaluated costs, with evidence of cost savings in one study."<br><br>"Home telerehabilitation showed improved function, strong satisfaction, travel and cost savings. Two studies in the present review included economic analyses. The cost-analysis study from Tousignant et al. was a multicenter RCT with a large sample. They noted early total cost savings when

			musculoskeletal disorders, including cost–benefit analyses.”		there was greater distance from in-person care, however, the economic analyses did not include cost-benefit analyses, which help to understand the cost impacts for the patient in addition to the health system. Kairy et al. (2009) advised that it is important to match cost savings with health outcomes.”<br><br>**Access**<br>“There are substantial barriers to access healthcare services in rural Canada compared with urban locations.1 In addition to higher injury rates, rural and remote residents are30% more likely to have chronic back disorders and arth-ritis.2,3 Reduced access to appropriate healthcare is thought to be a reason for higher rates of chronic health conditions in rural areas.4,5Physical therapy is an important component of the management of musculoskeletal disorders, yet it is no treadily available for rural and remote residents.6 For example, only 10% of physical therapists practice in rural Saskatchewan, Canada,7 while approximately 30%of the population lives in these

					regions." (comment background)
**17**	Hewitt (2020)<br>SR<br>N=19	Musculoskeletal Conditions<br><br>**Age**<br>"All studies included participants with an average age of 35 to 69 years."<br><br>**Gender**<br>"Of the studies that reported on the gender of participants (15 studies), all studies except 3 had a greater number of female participants."	This review demonstrated the positive impact of digital health interventions on musculoskeletal conditions, particularly in improving pain and functional disability. The evidence suggests benefits in coping strategies and catastrophizing. As the aging population grows, these interventions have the potential to alleviate personal, societal, and economic burdens.]	**NR**	**Cost**<br>"Treating musculoskeletal conditions is estimated to cost the United States US $213 billion and costs the UK economy £10.2 billion (US $12.62 billion) in direct costs to the National Health Service. Digital health interventions can provide high-reach, low-cost, readily accessible, and scalable patient education and self-management interventions that address time and resource constraints for musculoskeletal populations, delivered via apps or web-based platforms. This, in itself, is an important finding, as although not always superior to interventional controls (usual care), digital health interventions have the ability to deliver safe, high-reach, low-cost, readily accessible, and scalable care." (comments background and discussion)
**18**	Hosseiniravandi (2020)<br>SR<br>N=50	MS, ABI, SCI, Multiple neurological conditions, DS, stroke, COPD,	The examination of Home-Based TeleRehabilitation (HBTR) software systems for remote supervision of Home-Based	**NR**	**Cost**<br>"Patients go through a period of short-term illness rapidly and are discharged from hospitals faster than in

		cardiovascular system, CAD, Older adults<br><br>**Age and Gender**<br>NR	Rehabilitation (HBR) programs reveals common functionalities among these systems, offering the potential for comprehensive HBTR systems. Despite these promising features, research in this area is in its early stages. A deeper understanding of these systems could contribute to the optimal design of HBTR systems, resulting in enhanced HBR programs and streamlined remote supervision.		the past. This is mainly due to the time constraints and economic considerations faced by today's healthcare organizations. However, many of these patients still require rehabilitation services to completely recover from illness. Furthermore, such services are often associated with substantial costs and the patients sometimes have to make multiple trips to the rehabilitation center during the treatment process."<br>(comment introduction)
**19**	Jim (2019)<br>SR and MA<br>N=32	Coronary Heart Disease<br><br>**Age**<br>The mean age of participants was 61.72 (±4.26) years<br><br>**Gender**<br>"The majority (about 77%) of study participants were men although no trials excluded women, and in one study sex was not reported."	"Telehealth significantly improved cardiovascular risk factors. Although not significant, fewer deaths occurred over time with telehealth interventions. Telehealth could enhance access to formal cardiovascular secondary prevention and narrow the current evidence–practice gap."	**Adverse event**<br><br>"No trial reported any adverse<br>events as a result of participation in the programmes."	**Cost**<br>"Management of CVD creates substantial economic burden, with estimated costs of €210bn per year spent on CVD management in the European Union (EU).2 The majority of acute CHD admissions and events occur in those with previous CHD events,3and this is where the greatest economic burden lies, with 68% of total costs spent on secondary care.4 Therefore, to prevent CHD admissions and reduce costs, secondary prevention is of paramount importance and includes effective lifestyle risk factor reduction and prescription of, and adherence to,

					cardioprotective medications. Cardiac rehabilitation has evolved as the principal method of delivery of secondary prevention, and has been shown to reduce morbidity and mortality,5 to improve quality of life and to be cost-effective when compared with no cardiac rehabilitation." (comments introduction)<br><br>"Only three trials reported costs of delivery, with all three stating that the interventions cost less to deliver than usual care, but only two including a full cost-effectiveness evaluation.46,48 One trial showed telehealth intervention appeared to be cost-effective compared with usual care for increasing walking among CHD patients. The other study reported a net cost saving of US$965 per person with an estimated return of 213% on telehealth intervention."<br><br>**Access**<br>Telehealth interventions mostly delivered by phone and/or Internet could enhance access to a formal secondary prevention by

					patients unable to attend centre-based car-diac rehabilitation and could therefore narrow the current evidence–practice gap in this specific area. (comment conclusion)
**20**	Johansson (2011)<br>SR<br>N=9	Stroke<br><br>**Age**<br>NR<br><br>**Gender**<br>One study reported 35 men and 39 women and their primary family caregivers (7 men and 67 women)			**Cost**<br>"No study reported information on cost effectiveness or on resource utilization. Some studies mentioned investment costs."<br><br>**Access**<br>"An Internet-based educational intervention aimed to support stroke caregivers living in rural communities. The participants were linked to a customized educational care website giving 'tips of the month' and educational information."<br><br>**Digital Literacy**<br><br>One study had the objective of exploring the feasibility of providing Internet-based education and support to caregivers.
**21**	Kebapci (2020)<br>SR<br>N=15	Cardiac conditions			**Cost**<br>"Advantages to eHealth include an individualized learning environment at a

		**Age and Gender**<br>NR			lower cost than hospital-based interventions."<br><br>**Socioeconomic and educational level**<br><br>"Poor adherence has been associated with those with lower educational level, lack of social support, high burden of family responsibilities, economic challenges, lack of or limited healthcare insurance, and lower age (<65 years)." (comment introduction)
**22**	Knepley (2020)<br>SR<br>N=34	Stroke<br><br>**Age and Gender**<br>NR	TR emerges as an equally effective alternative to conventional clinic-based rehabilitation for enhancing functional outcomes post-stroke. Patient satisfaction with TR varies but offers advantages like enhanced accessibility and potentially reduced costs, making it a valuable option for those facing financial or logistical constraints. TR's flexibility is evident through home-based robotic therapy, speech therapy, and virtual reality applications. Moreover, TR can be seamlessly integrated with clinic-	**NR**	**Cost**<br><br>"Two studies addressed cost: Llore´ ns et al. found VR-based TR to cost $654.72 less per patient than clinic-based rehabilitation, a savings of 30.5%over the course of the program.13 Housley et al. reported a cost savings of 64.97% for home-based robotic therapy over clinic-based therapy."<br><br>**Access**<br>"Although some patients prefer the gratification of direct human interaction, other patients may favor the increased convenience and accessibility of TR over

			based rehabilitation or other services like home visits, thereby improving patient compliance and expanding its scope in diverse rehabilitation strategies.		repeated clinic visits. No significant differences in functional outcomes were shown between patients who used community-based TR and patients who exercised at home without TR."<br><br>**Digital Literacy**<br>"Although improving access for those who lack home internet access or internet literacy, community-based TR has apparently lower compliance than home-based TR."
**23**	Pastora-Bernal (2017)<br>SR<br>N=15	Orthopedics conditions who had surgery<br><br>**Age**<br>Adults (≥18 years<br><br>**Gender**<br>NR	"Conclusive evidence on the efficacy of TR for treatment after an orthopedic surgery, regardless of pathology, was not obtained. We found strong evidence in favor of telerehabilitation in patients following total knee and hip arthroplasty and limited evidence in the upper limb interventions (moderate and weak evidence). Future research needs to be more extensive and conclusive."		**Cost**<br>"Economic and cost-utility outcomes were not analyzed, nor were patient and clinician satisfaction or those outcomes measuring adherence to, or compliance with, rehabilitation programs."<br>"The growing demand for rehabilitation can result in increased costs and longer waiting lists, becoming a threat to the sustainability of health care services. Telerehabilitation can help with this issue by discharging patients from points of care while improving their adherence to treatment." (comment introduction)

**24**	Pietrzak (2013)<br>NR<br>N=5	Osteoarthritis<br><br>**Age**<br>60 years or older<br><br>**Gender**<br>NR	Internet-based technologies are increasingly employed for health services, particularly in information, education, and medical service delivery. This review underscores the successful utilization of the Internet for community-based self-management and rehabilitation interventions in OA, leading to improved health indicators, enhanced access to care, and better communication between OA patients and health professionals. High patient satisfaction with Internet-based OA interventions is evident. The findings emphasize the importance for healthcare providers managing OA to strategize the effective use of Internet-based technologies to mitigate the disabling effects of joint degeneration in the OA population.	**Handling data/data protection/data security**<br><br>"The concerns surrounding the of security of patient data are one of the main problems inhibiting the introduction of e-health services in Australia. At present, desktop videoconferencing programs that offer secure video rooms are available at a low cost ($35/month plus 6¢/min).32 The security is provided by using 128-bit AES encryption for data in transit and unique URLs for each of the online meetings to ensure that only authorized people are able to access the Web conferences. Finally, an alternative way of ensuring patient data security is to avoid sending sensitive information over the videoconferencing network."	**Cost**<br>"However, the computer and connection requirements were low: a conventional personal computer, low-bandwidth Internet Protocol (18 kiolbits/s) via standard modems, and low-cost Web cameras to facilitate a real-time video and audio connection were used."
**25**	Saragiotto (2016)<br>SR<br>N=37	Low back pain<br><br>**Age and Gender**<br>NR	"Motor control exercise seems to be more effective than a minimal intervention for reducing pain, but does not have an important effect on disability, in patients with chronic LB. There is no clinically important	**NR**	**Cost**<br>"Trials including cost-effectiveness analysis and long-term outcomes are also needed in this area."

			difference between motor control exercise and other forms of exercises or manual therapy for acute and chronic LBP. The choice of exercise for chronic LBP should depend on patient or therapist preferences, therapist training, costs, and safety. We are uncertain about the effectiveness of MCE compared with exercise and EPA, as the quality of the evidence is low or very low. It is unclear whether motor control exercise can prevent recurrences of low back pain because the evidence was of very low quality."<br>"There is very low to low quality evidence that MCE is clinically more effective than exercise and electrophysical agents (EPAs) or telerehabilitation for pain and disability."		
**26**	Schroder (2019)<br>SR<br>N=7	Stroke<br><br>**Age**<br>47 to 76 y<br><br>**Gender**<br>NR	TR holds promise in overcoming accessibility challenges to rehabilitation in the future. Limited evidence suggests that VR based therapy is motivating, allowing for extended exercise sessions and increased adherence to therapy. Combining the accessibility of tele-	**NR**	**Cost**<br>"Indeed, VR-based rehabilitation has been found to be feasible and effective in patients after stroke, for example, by the use of the Nintendo Wii which is affordable and accessible suggesting its potential for home-based training."

			rehabilitation with the motivational aspects of VR appears convenient, as highlighted in the current review. Functional improvements achieved through this approach are comparable to similar interventions supervised by therapists in clinics, indicating the potential for efficient rehabilitation at home. However, it is crucial to note that this should not replace current practices but enhance the efficient use of resources and promote more active exercise time.		"Cost–benefit. The factors which highly influence the costs of stroke rehabilitation are human resources and travel expenses of the patients. Regarding those factors, Llorens et al. found, by comparing home-based and clinic-based groups, that if parts of rehabilitation can be transferred to the homes, human resources can be used more efficiently and costs of transportation services can be lowered. A similar cost-effective use of human resources is reported by Kripic et al. However, tele-rehabilitation required specific equipment which can be cost-demanding."
**27**	Shukla (2017)<br>SR and MA<br>N=6	Total knee arthroplasty<br><br>**Age**<br>Ranged from 66 to 73.3 year<br><br>**Gender**<br><br><br>Piqueras (2013): Female Percentage: 72.4%<br>Male Percentage: 27.6%<br>Tousignant (2013)14:	"The patients in the home telerehabilitation group showed improvement in physical activity and functional status similar to patients in the conventional therapy group. This systematic literature review has demonstrated that the home telerehabilitation method is an acceptable rehabilitation method for patients who underwent TKA. Telerehabilitation seems to be a practical alternative to conventional face-to-face rehabilitation	**NR**	**Cost**<br>"Cost-effectiveness evaluations should also be planned alongside the clinical trial to provide a more holistic view to the healthcare providers about the viability of this alternative to the conventional therapy for implementation."(comment discussion)

		Female Percentage: 41%<br>Male Percentage: 59%<br>Russell (2011)15:<br>Female Percentage: 61%<br>Male Percentage: 39%<br>Kramer (2003)13:<br>Female Percentage: 59%<br>Male Percentage: 41%<br>Cabana (2010)17:<br>Female Percentage: 55%<br>Male Percentage: 45%<br>Russell (2004)18:<br>Female Percentage: 46.7%<br>Male Percentage: 53.3	therapy inpatients who underwent TKA."		
**28**	Simek (2012)<br>SR and MA<br>N=23	Older adults at risk for falls	"The adherence of older adults to home exercise programs for the	**NR**	**Cost**<br>"Providing additional support increases the cost of

		**Age**<br>older or equal to 60 years of age<br><br>**Gender**<br>NR	prevention of falls is low and may be affected by specific program characteristics. Higher levels of full adherence were found in interventions containing balance or walking exercise, moderate home visit support, physiotherapist led delivery and no flexibility training. Higher levels of partial adherence were found in interventions containing home visit or telephone support, a participant health service recruitment approach and no group exercise training. There was an absence of evidence to link adherence to intervention efficacy for the prevention of falls."		exercise program prescription. Thus further research is indicated to examine the cost-effectiveness of providing home visit or telephone support so that economically efficient prescription approaches can be identified."
**29**	Slattery (2019)<br>SR and MA<br>N=30	Chronic pain (nonspecific back pain, chronic lower back pain, multiple pain, migraine or headache, fibromyalgia, MSK pain, arthritis, chronic no n cancer pain, non specific low back pain.	"This systematic review with an NMA generated comparisons between eHealth modalities previously not compared to determine which delivered the most effective interventions for the reduction of pain interference in chronic pain patients. There are limitations with this review, in particular, the underrepresented nature of some eHealth modalities included in the analysis.	**Autonomy**<br>"Self-management of chronic/long-term illnesses through education and supportive interventions can not only decrease utilization of health care services but may also lead to improvements in clinical outcomes and overall quality of life."	**Cost**<br>"Empowering individuals to take an active role in their own health care has been identified as a crucial factor for improving the quality of care and reducing health care costs."<br><br>**Access**<br><br>"Web-based eHealth intervention may improve accessibility to treatment, reduce the waiting list duration, and can be

		cancer-related chronic pain)<br><br>**Age**<br>36.7 to 65.8<br><br>**Gender**<br>More than 60% of females in 24 studies	However, in the event that the review is regularly updated, a clear ranking of eHealth modalities for the reduction of pain interference will emerge.”		delivered more cost-effectively than in-person service.”
**30**	Speyer (2018)<br>SR and MA<br>N=44	**Morbidity**<br>NR<br><br>**Age**<br>Children were the target population in 10 (23%) studies, and adults were the target in 33 (76%) studies; with 1 (2%) study having a population of both adults and children.<br><br>**Gender**<br>NR	“Telehealth services may be as effective as face-to-face interventions, which is encouraging given the potential benefits of telehealth in rural and remote areas with regards to healthcare access and time and cost savings.”	NR	**Cost**<br><br>“Further research is required to understand the most effective uses for telehealth in relation to quality of healthcare, access to services, cost-savings and identifying strategies to improve the effectiveness and sustainability of telehealth services.”<br><br>**Access**<br>“In particular, since individuals living in rural and remote areas may have limited access to face-to-face treatment, telehealth may offer allied health services at higher frequencies over a longer period compared with face-to-face interventions.”<br><br>**Ethnicity**<br>“In 7 (16%) studies, the nationality of the study site

					or participants was not clearly reported, although the authors indicated that the study was conducted in a rural area.”
**31**	Srikesavan (2019)<br>SR<br>N=4	Rheumatoid arthritis<br><br>**Age**<br>49.5 to 69.3<br><br>**Gender**<br>The proportion of female participants ranged from 29%to 93% in web-based intervention groups and from 77%to 95% in control groups.	“The available evidence on web-based interventions for people with RA seems to indicate that there is a viable space for web-based interventions; however, to date, they are not adequately developed or evaluated to recommend their use in people with RA. Fully powered trials with theory-based and patient centred interventions and with long-term follow-ups are needed.”	**NR**	**Cost**<br><br>“There is further need for adequately powered randomised controlled trials to evaluate clinical and cost-effectiveness of web-based interventions in people with RA.”
**32**	Tchero (2018)<br>SR and MA<br>N=15	Stroke<br><br>**Age and Gender**<br>NR	“Telerehabilitation can be a suitable alternative to usual rehabilitation care in poststroke patients, especially in remote or underserved areas. Larger studies are needed to evaluate the health-related quality of life and cost-effectiveness with the ongoing improvements in telerehabilitation networks.”	**Autonomy**<br>**“**Compared with usual rehabilitation, telerehabilitation offers several advantages, including easier access, mentoring for disabled stroke patients, and the ability of patients to self-record on their pain, mood, and activity.”	**Cost**<br><br>“Lloréns et al reported data on the cost-effectiveness outcome. They calculated that for 1 participant, the cost of telerehabilitation was lower than that of usual care by about US $654 (US $1490.23 and US $853.61 for in-clinic and home rehabilitation programs, respectively). Although setting the virtual reality system at home required US $800, the telerehabilitation arm required fewer work hours by physical therapists

					and it eliminated the cost of round trips to the clinic with every session."<br><br>**Access**<br>"Telerehabilitation can be a suitable alternative to usual rehabilitation care in poststroke patients, especially in remote or underserved areas."
**33**	Van der Meij (2016)<br>SR<br>N=27	Postoperative (cardiac, cancer, gynaecological and orthopaedic surgery)<br><br>**Age**<br>participants varied from 43.2 years to 75.3 year<br><br>**Gender**<br>Most studies included both male and female patients, except for one study which included patients undergoing gynaecological surgery.	"Based on this systematic review we conclude that in the majority of the studies e-health leads to similar or improved clinical patient-related outcomes compared to only face to face perioperative care for patients who have undergone various forms of surgery. However, due to the low or moderate quality of many studies, the results should be interpreted with caution."	**Adverse event**<br>"In this assessment, five items were scored by a Perioperative E-Health Interventions by a notably low number of studies: if an assumption was made to blind the patients or the caregivers, whether adverse events were being reported, if compliance with the intervention was reliable and if the study had sufficient power to detect a clinically important effect. We considered all five items to be important risk factors for introducing bias Although, we understand that blinding of the patients and caregivers is difficult in this type of studies, measuring the compliance and adverse events should be an integral part for this type of research. In addition, the fact that only	**Cost**<br><br>"11 studies (40.7%) reported outcome measures related to the effectiveness of the intervention in terms of health care usage and costs. "<br><br>"Five out of six studies reported on costs related to direct and indirect health care costs. The majority (n = 4) included the extra costs for the intervention. Only one trial reported the cost-effectiveness of the intervention calculating the ICER related to the effect on physical activity. There was also a trial which only reported the estimated cost savings based on the length of stay in hospital. Only two studies reported a positive

				nine studies performed a power calculation and included enough patients, requires to interpret the results of this review with caution.”	effect in costs. For one such study the effect depended on the travel distance for the patient between their residence and the hospital. For the other three studies, differences in costs were measured between the two groups. All were large studies (at least 147 participants) but with a high or medium risk of bias.”
**34**	Van Egmond (2018)<br>SR and MA<br>N=23	Surgical patients<br><br>**Age**<br>mean 43.2 to 87.1<br><br>**Gender**<br>Overall 56% females	“Physiotherapy with telerehabilitation has the potential to increase quality of life, is feasible, and is at least equally effective as usual care in surgical populations. This may be sufficient reason to choose physiotherapy with telerehabilitation for surgical populations, although the overall effectiveness on physical outcomes remains unclear.”	**Adverse event**<br>One study had adverse event as an outcome.<br><br>**Autonomy**<br>Four studies included self-efficacy outcomes	**Cost**<br>“Over the last few years, telerehabilitation services have developed rapidly, and have the potential to be a more cost-effective alternative for outpatient assessment and treatment in hospital due to the ability to reach people in remote areas or at home. Despite methodological shortcomings within the included studies, this review illustrates the feasibility of telerehabilitation in surgical patients, but research relating to the (cost-) effectiveness and patient satisfaction of telerehabilitation requires further exploration.” (comment introduction)<br><br>**Digital divide/ disability**<br><br>“A variety of inclusion and exclusion criteria have been

					reported. The majority of studies included patients who were discharged home or to short-term rehabilitation, who had a telephone or access to high-speed internet services, and had no communication disorder.” (Our comments to this review)
**35**	Velayati (2020)<br>SR<br>N=8	Older adults use of TR after surgery (Stroke, COPD, TKR, Chronic heart failure)<br><br>**Age**<br>60 or older<br><br>**Gender**<br>NR	“In this study, several papers were reviewed to compare the effectiveness of telerehabilitation interventions with the traditional rehabilitation approaches for therapeutic pur- poses in the elderly. The results indicated that a wide range of simple and complex telerehabilitation interventions were used. Overall, in most studies, there was no significant difference between the intervention and control groups and the level of improvements was similar for most out- comes. The results of this study provide evidence for regard- ing telerehabilitation services as an alternative to traditional rehabilitation approaches to reduce outpatient resource utilization and improve quality of life. “	**Autonomy**<br>2 studies included self-efficacy outcome and one study included a web portal with a symptom diary for self-treatment of exacerbation outcome.	**Access and cost**<br><br>“Telerehabilitation is an ex- ample of using ICT in the field of rehabilitation that saves time and costs and aims to provide the vulnerable populations, for example, the elderly and disabled people with easier and continued access to healthcare services.” (comment introduction)<br><br>**Digital literacy/ Digital Divide**<br><br>“It seems that the accessibility of different types of technology, strong information technology infra- structure, and the high level of patients’ computer literacy are the main reasons for paying more attention to tele- rehabilitation technology in the developed countries.”

					**Digital divide/access to technology**<br><br>“The effectiveness of telerehabilitation interventions could be related to the accessibility of telerehabilitation equipment at home.” (comment discussion)
**36**	Veras (2017)<br>Scoping Review<br>N=28	Stroke<br><br>**Age**<br>NR<br><br>**Gender**<br>177 males and 133 females. Seven studies (25%) did not report the sex of the patients	The review emphasizes the growing evidence supporting the efficacy of VR and TR in enhancing stroke care. It provides guidance on selecting outcome measures to assess the impact of these interventions on stroke survivors and their families. The use of reliable and comparable tools is crucial for research and rehabilitation care to evaluate the quality of care, identify gaps, and prioritize stroke care elements such as functioning, cognition, participation, quality of life, professional training needs, and resource utilization. The identified outcome measures focus on motor, sensory, and other body functions. The review suggests that future studies in VR and TR should incorporate existing standardized outcome	**Adverse event**<br>“The benefits of using standardized outcome measures include identifying patients who are at risk for poor or adverse out-comes,4 determining the most effective interventions in specific contexts and assessing organizational performance.” (comment introduction)<br><br>“Other evaluation gaps identified in this review were related to changes in health service use, cost of the interventions and the use of specific tools to assess patient safety, comfort, simplicity of use, effectiveness and adverse consequences due to the interaction with the technology.” (comments on discussion)	**Cost**<br>“Likewise, most of the descriptions of the technologies did not report information about the costs of the interventions, which could help providers, clinicians, patients and families to make decisions based on the key outcome domains and costs of the intervention. The studies did not conclude information about costs, although more recent studies are starting to report costs of the interventions.”<br><br>**General equity comment**<br>“In general, most of the studies lacked information on participant characteristics such as age, sex, education and economic status. This information is valuable to identify differences in health outcomes and access to VR and TR services” (comment on results section)

			measures covering various impacts and components of the International ICF model. This approach enhances the comparability of research findings at national and international levels, supporting the evaluation of intervention impact in stroke rehabilitation at both individual and community levels.		
**37**	Wake (2020)<br>Scoping review<br>N=77	Trauma (TBI, SCI and multiple trauma)<br><br>**Age**<br>Adults (≥18 years old) accounted for 49.4% (n= 38) of the participants, whilst paediatrics (<18 years old) accounted for 41.6% (n = 32). Of the remaining seven (9%) studies, ages were either unclear or contained both adults and paediatrics.<br><br>**Gender**	"This review demonstrates how telehealth is utilised across a spectrum of patients with traumatic injuries and to facilitate delivery of therapy, specialist consultations and assessments, with many studies reporting improvements to health. There is a paucity of high-quality rigorous research, which makes replication of findings and uptake of the intervention problematic. Future telehealth and trauma research should focus on the quality and reproducibility of telehealth interventions and the economic feasibility of using this platform to deliver trauma care."	NR	**Cost**<br>"In relation to costs and resources, using telehealth was found to reduce the number of transfers between hospitals, in some cases by 73%.64 Financial savings were reported, ranging from € 365 per transfer up to a total saving of € 3.5 million over 14 years."<br><br>**Access and cost**<br>"It is also possible that the limited number of specialist paediatric rehabilitation services (relative to adult services) are generally provided in major metropolitan areas, requiring extended time away from home, disruption to families and financial cost."

		64% males			
**38**	Wang (2019)<br>SR and MA<br>N=21	Total knee or hip replacement for people with osteoarthritis<br><br>**Age**<br>Mean=65.2<br><br>**Gender**<br>13 studies had more than 50% females participants, 7 studies had less than 50% of females participants and one study did not reported it.	"There is moderate-quality of evidence showed technology-assisted rehabilitation, in particular, telerehabilitation, results in a statistically significant improvement in pain; and low-quality of evidence for the improvement in functional mobility in people undergoing TKR. The effects were however too small to be clinically significant. For THR, there is very limited low-quality evidence shows no significant effects."		**Access**<br><br>"When participants were asked what they liked most about the application, no travelling to the hospital was cited by 57% and ease of access by 21%." (study results)<br><br>"Compared to face-to-face rehabilitation, services delivered remotely via telephone or internet are more affordable and accessible, particularly for people living in rural areas." (comment introduction)<br><br>"Given the fact that technology could improve the healthcare accessibility and treatment adherence, despite its clinical effectiveness was similar comparing to conventional intervention, it still has a very promising role in circumstances when access and adherence are challenging." (discussion comment)
**39**	Ben Bowhay (2023)<br>N=8	Cystic Fibrosis (CF)<br><br>**Age**<br>6 to 51 years; Some studies	"The current review highlighted promising CFQ-R improvements within the respiratory and physical domains following 6–12 weeks of	**Adverse events**<br><br>"No adverse events were reported across all studies"	**Access**<br><br>"Telemedicine enables pwCF to access exercise programs while

		don't specified age<br><br>**Gender**<br>77 M, 93 F, one study don’t report sex/gender	telemedicine-based exercise. However, no significant changes were found in ppFEV1 outcomes. This suggests that pwCF may find telemedicine-based exercise beneficial for enhancing their QoL, despite having no clear pulmonary function improvements.”	“Moreover, only one study in our review met the WHO physical activity recommendations with over 75 minutes of vigorous intensity physical activity performed weekly. This suggests that the majority of interventions may not have been adequate to promote pulmonary function, and therefore, this intensity (or lack thereof), alongside the impact of intervention length, and the aforementioned variation in study design, means we cannot determine the efficacy of specific exercises at present. Therefore, longer study durations with rigorous follow-up may be required for telemedicine-based exercise interventions with people with CF.”<br><br>**Privacy / patient identification**<br><br>"…one study did not report individual patient data for the assessed outcomes due to the risk of participant identification"	conforming with infection control requirements and preventing cross-infection, a requirement that restricts group exercise participation” (comment introduction)
**40**	Hao<br>(2023)<br>SR & MA	Stroke<br><br>**Age**	“Virtual reality-based telerehabilitation is a promising avenue for	**Adverse events**<br>“Three studies reported there was no intervention-related	**Costs**<br>“Effective and efficient rehabilitation programs

	N=9	“participants were adult patients (age >= 18 years old)” (No details)<br><br>**Gender**<br>NR	patients with stroke, given the barriers and restrictions of traditional in-person rehabilitation. The combination of virtual reality and telerehabilitation technologies is feasible in stroke rehabilitation. Compared to in-person rehabilitation, virtual reality-based rehabilitation demonstrates comparable outcomes in upper extremity function, and equivalent effects on balance ability. Although delivered remotely, patients might still have a similar subjective experience of rehabilitation with therapists’ supervision as in-person rehabilitation. virtual reality- based telerehabilitation has the potential to become the economically favorable rehabilitation delivery model, even though the direct evidence on this area is spare and warranted”	adverse event.” (Not specified for the others)<br><br>**Autonomy**<br><br>“These stroke survivors suffer from a variety of impairments, including motor, sensory, speech, cognition, and psychosocial aspects. Those impairments affect their independence to perform daily activities, and furtherly hamper their participation and quality of life. Effective and efficient rehabilitation programs could augment functional recovery, mitigate activity limitation, prevent secondary impairments and reduce **subsequent costs**.” (introduction comment)<br><br>**Participation**<br><br>“The effects of virtual reality in stroke rehabilitation have been identified in multiple dimensions of functional recovery, including balance, gait, mobility, upper extremity function, cognitive ability, **activities of daily life function** and **participation**.” (introduction comment)<br><br>**Handling data/data protection/data security**	could augment functional recovery, mitigate activity limitation, prevent secondary impairments and reduce subsequent costs.” (introduction comment)<br><br>“According to the American Stroke Association, telerehabilitation has the potential to provide timely and efficient care to post-acute stroke survivors beyond the hospital and into the home, improving patients’ functional outcomes while reducing long-term disability and costs.”<br><br>“Only one study in this review conducted the cost-benefit analysis. Llorens et al. compared the cost of virtual reality-based telerehabilitation to the conventional approach that had virtual reality sessions in the clinic, indicating the in-clinic intervention led to on average $654.72 per person more expense than the virtual reality-based telerehabilitation approach. Human resources (e.g. hours of physical therapists) and transportation were the main difference between the two groups in cost” (Results section)

				The virtual reality system was installed at patients' locations and the data from training sessions could either be shared simultaneously with therapists through the internet or stored offline for further performance analysis.	"The cost-effectiveness of telerehabilitation services in stroke rehabilitation is still lacking and more studies are warranted to examine the economic implications of virtual reality-based telerehabilitation as the system installment cost could be a financial burden for patients and clinical settings" (Discussion section)<br><br>"The cost-effectiveness of virtual reality-based telerehabilitation was positive, but limited to one study, which found that telerehabilitation induced less cost due to the saving on human resources and transportation while taking account of the expense of instrumentation of a virtual reality system" (Discussion section)<br><br>**Urban-rural**<br>"In rural areas, due to limited resources and accessibility, the rehabilitation demand of stroke survivors is often unmet, and the optimal outcomes cannot be achieved." (introduction comment)<br><br>**Social distancing**

					“Telerehabilitation provides an alternative possibility to deliver rehabilitation services via up-to-date health technology. It plays a critical role in the midst of the global pandemic while conventional in-person rehabilitation sessions are affected by **social distancing restrictions**.” (Discussion section)
**41**	Mahmood (2023) Scoping review N=72	Stroke, musculoskeletal injury, low-back pain, bipolar disorders, osteoarthritis, osteoarthritis knee, schizophrenia, kidney transplant recipients, sport injuries, hypertension, diabetes, obesity, chronic musculoskeletal pain, multiple sclerosis<br><br>**Age**<br>Not reported, but<br>2 studies about older adults/elderly people	“Exercise adherence is crucial for recovery and successful implementation of exercise interventions. Adherence is a complex and multifactorial phenomenon that is less researched. To date, there are very few reliable measures of exercise adherence. The interventions that support adherence are varied and incorporate behavioral modifications. However, specific interventions for different disease populations are not yet established.”<br><br>(Note: findings in the study about telerehabilitation in multiple sclerosis)<br>“Baseline patient information is important for individualizing		**General equity**<br><br>“Future studies should investigate the use of real-time adherence measures and evaluate the effectiveness of strategies that target factors of nonadherence at an individual, interpersonal, health system, and society level.” (Conclusion)<br><br>**Digital Literacy**<br>“Some other barriers of adherence are cultural/language barrier, miscommunication between healthcare provider and patients, lack of supervision by healthcare provider, poor health literacy, patients' readiness to change, presence of cognitive impairment, cost, and access to treatment centers”

		**Gender**<br>Not reported	telerehabilitation and eliciting active participation”		**Costs**<br>“Telerehabilitation and mHealth were also deployed in various studies … since it provides enhanced education and awareness about diseases, has more control over risk factors and has lower health cost.”
**42**	Munoz-Tomas (2023)<br>SR<br>N=11	Cardiac pathology, Neurologic pathology, Breast cancer, Musculoskeletal pathology; total knee arthroplasty; MS, chronic low back pain<br><br>**Age**<br><br>“All studies included participants over 18 years old, with the average age being 57.35 in the TR group and 59.19 in the control group”<br><br>**Gender**<br>“In terms of gender, there were women	“Most of the studies concluded that physiotherapy interventions using telerehabilitation were at least as effective as traditional rehabilitation interventions and are considered feasible and effective options. In general, telerehabilitation interventions were shown to improve functional level and quality of life, as no significant differences with control groups were observed. This review provides information on patients’ high levels of satisfaction and adherence, with values equivalent to traditional rehabilitation in all cases. However, such variable was not considered in all cases. Another conclusion drawn from our work is that there are few studies in which telerehabilitation is used to	**Autonomy**<br><br>“Telerehabilitation can help improve quality of life and activities of daily living although supervised exercise without TR may be more beneficial for fatigue and health profile.” (Result in one study)	**Cost**<br><br>“The advantages of lower cost and less interference of rehabilitation processes in patients’ daily lives, according to the report of Stefanakis et al., indicate that the risk of adverse effects seems very low and could justify the implementation of telerehabilitation in controlled clinical settings” (Discussion)<br><br>“Efficient and cost-effective alternative, individualized intervention and overcoming accessibility barriers, implementation costs of the REMOTE-CR program were substantially lower than those of CBexCR” (Result in one study)<br><br>“Lower costs in TR, fewer rehospitalizations than in

		participating in all studies, with a sample size of 100% in a study of breast cancer survivors. Women's smallest representation was 14.2%, in a study on coronary heart disease."<br><br>Note: excluding the study about breast cancer (76F), 594M (54%) – 509F (46%)	apply therapeutic exercise. This shows that we have not really learned from the time of the pandemic, when different telerehabilitation tools were generally implemented in the treatment process. The results of this study show that prescribed telerehabilitation exercise is as effective as face-to-face exercise, and can set a basis for possible telerehabilitation practices in a post-pandemic world."		GC, no inferiority in terms of knee flexion–extension and gait speed." (Result in one study)<br><br>"Regarding satisfaction levels, in the case of patients with musculoskeletal disorders who received telerehabilitation and face-to-face rehabilitation, results were similar for both groups. The studies that analyzed and compared the costs of both types of intervention for patients with this condition reflected lower overall costs for the TR group." (Results / Pathologies Studied)<br><br>"Maddisson et al. [25] also took treatment adherence and cost-effectiveness into account. In both studies, regarding functional level, treatment with TR was equally effective as in-person treatment in the physiotherapy room. Quality of life was satisfactory in both groups. Adherence was higher in the TR group, while costs were significantly lower in the TR group" (Results / Pathologies Studied)<br><br>"Prvu Bettger et al. [27] showed that

					telerehabilitation in patients undergoing knee replacement surgery had lower total costs when compared to traditional rehabilitation. Other authors, such as Maddison et al. [25], have shown the beneficial cost-effectiveness of the REMOTE-CR program in patients with coronary heart disease. In addition, reduction in drug costs through individualized interventions was observed, as well as benefits due to overcoming accessibility barriers." (Results / Limitations of the Studies Regarding the Use of Telerehabilitation)<br><br>**Urban-rural**<br><br>"Some of the studies referred to certain limitations when using telerehabilitation. Chen et al. [20] reported that, positively, home-based telerehabilitation may be an important way of overcoming barriers and may be useful for stroke survivors living in rural areas. Although cost-effectiveness was not observed, it is likely to reduce **costs** and travel time" (Results / Limitations of the

					Studies Regarding the Use of Telerehabilitation)<br><br>"When focusing on where telerehabilitation was carried out, Hwang et al. [22] indicated that their study was conducted in a metropolitan area with good Internet connection. In addition, they reported that further research would be needed to determine the applicability of telerehabilitation in rural and remote areas with unsteady Internet connection." (Results / Limitations of the Studies Regarding the Use of Telerehabilitation)<br><br>**Access**<br>"Easy access for the population, high levels of satisfaction, physical and functional results not inferior to those obtained with traditional physical therapy. Program compliance rate in favor of TR." (Result in one study)

Notes: ABI=acquired brain injury; ADLS= activities of daily living; AR=augmented reality; DS= Down's syndrome; CVD= cardiovascular disease; CR= cardiac rehabilitation; CAD= coronary artery disease; COPD=Chronic Obstructive Pulmonary; F=Female(s); ICF= Classification of Functioning, Disability, and Health; ICT= information and communication technology; OCER=incremental cost-effectiveness ratio; M=Male(s); MS= multiple sclerosis; MSDs=musculoskeletal disorders; NDTs= neurodevelopmental techniques; NDT= Neurodynamic test; NRS=Narrative Review Study; OR=osteoarthritis; PD= Parkinson's disease; PR=

Pulmonary rehabilitation; RA=Rheumatoid arthritis; RCT= randomized controlled trials; ROM= Range of Motion; SOTs= sensory organization tests; SCI= Spinal Cord Injury; TMed= telemedicine; VC= Virtual care; VR= virtual reality; TR= telerehabilitation;
