# Supplementary material for: A Rapid Review of Ethical and Equity Dimensions in Telerehabilitation for Physiotherapy and Occupational Therapy
Source: Int J Environ Res Public Health. 2025 Jul 9;22(7):1091. doi: 10.3390/ijerph22071091 (PMC12294586; doi:10.3390/ijerph22071091)
Supplement: Supplementary file 1 [file ijerph-22-01091-s001.zip › Table S3- Occupational therapy study characteristics (N-09) Apr 02 2025.pdf]

Table S3: Occupational therapy study characteristics (N=09)

Study#	Study Author<br>Year<br>Study Design<br>N# included studies	Participants<br>Morbidity<br>/Population<br>Age<br>Sex	Key findings	Ethical principles	Equity aspects
**1**	Angela (2019)<br>SR<br>N=10	MS<br>**Age and Gender**<br>NR	"Considering the complex impact of fatigue in MS, it is necessary to provide a multidisciplinary rehabilitative approach that includes the role of OT. In particular, the efficacy of occupational interventions requires the adoption of fatigue self-management programs to teach patients ways of managing daily fatigue and energy conservation programs."	**Autonomy**<br>"A recent meta-analysis that compared 8 RCTs focused on educational interventions, including self-management components (e.g., clients selecting strategies to manage fatigue based on their needs, environment, or preferences) (comments discussion)<br><br>" The authors Yu and Mathiowetz reported that adapted equipment, training in self-care, and occupation-based therapeutic activities with functional training, such as ADL training, can improve Functional Independence Measure and fatigue."<br><br>"The author reported limited evidence about online fatigue self-management course	**Cost**<br><br>" The supervised exercise sessions incorporate CBT, for example, setting goals, finding social support, and understanding the costs and benefits of exercise." (comment on discussion regarding an included study)

				(during 7 weeks) in a teleconference. They reported a significant reduction in fatigue impact, but this improvement was no better than that attained by face-to-face programs.”(comments discussion)<br><br>“In particular, the efficacy of occupational interventions requires the adoption of fatigue self-management programs to teach patients ways of managing daily fatigue and energy conservation programs.” (comments conclusion)	
**2**	Guay (2017)<br>SR<br>N=12	Caregivers of older adults (Dementia, cancer, TBI, stroke)<br><br>**Age**<br>NR<br><br>**Gender**<br>“Caregivers were mainly female adults or older adults and generally were either a spouse or a child of the care recipient.”	“ The findings from this systematic review suggest that Internet-based interventions with tailored behavior change content that are interactive, provide human support either by professionals or peers, and incorporate BCTs, such as provision of specific instructions regarding the behavior, problem solving, and stress management, can have positive effects on the psychological well-	NR	**Cost**<br>“ Internet-based interventions can thus offer an easily accessible alternative and can be more cost-effective than traditional face-to-face interventions. asynchronous communication can cost less to developers and be used more easily by caregivers than complex synchronous communication modes, such as videoconference. Isolation of the effects of

			being of caregivers of older adults. Further randomized controlled trials that demonstrate the effect of each component individually with appropriate control conditions, analyze their outcomes considering adherence to protocol, and structure their report according to reporting guidelines in eHealthare needed to strengthen the validity of these results.”		each component could help future research provide better cost-benefit analyses because some components require more resources than others to develop.” (comment)<br><br>**Access**<br>“ Having rapid and remote access to ahealth professional for advice and tailored support has been reported in previous studies of Internet-based interventions for care recipients as the primary factor predicting adherence”(comment discussion)<br><br>“ Knowing that they have access at any time and place to professional support may also make caregivers feel less worried.”<br><br>**Cultural context (support)**<br>“In a study comparing a chat group to a video support group for caregivers of people with Alzheimer disease, caregivers reported feeling more at ease on the video group and experienced a more natural

					communication with the other caregivers, despite technical difficulties.”
**3**	HungKn (2019)<br>SR<br>N=15	Hemiplegic cerebral palsy, stroke, TBI, breast cancer, tetraplegia, older adults, orthopedic issues, autism spectrum disorder, visual and fine motor deficits.<br><br>**Age**<br>Range: two years and six months to over 70.<br><br>**Gender**<br>NR	“Telerehabilitation offers an alternative service delivery model for occupational therapy, not only bridging distance but also offering user-friendly treatment for patients at home.”	NR	**Cost**<br>“ TR also include tele-evaluation in OT, such as the use of a low-cost traditional telephone system for conducting phone interview as an alternative for cognitive assessment, wheelchair and assistive device prescription and home modification, etc.”<br><br>“Previous review in the effectiveness of TR in relation to various pathologies and impairments, healthcare utilisation and cost of rehabilitation use have found that TR has similar clinical outcomes to face-to-face rehabilitation services.” (comments)<br><br>**Access**<br>“It is becoming increasingly evident that TR can improve access to rehabilitation services, prevent unnecessary delays in the receipt of care.”<br><br>“Long travel time and distance, complicated service structure and the cost of intensive clinic-

					based interventions hinder clients' access." (comments Introduction)
**4**	Ninnis (2019)<br>Scoping review<br>N=14	Stroke, children with learning difficulties, cognitive impairment, MSK<br><br>**Age**<br><br>Atwal et al. (2013), Money et al. (2011) UK:<br>Participants (occupational therapists): Female: 80%; Age: Not reported.<br>Atwal et al. (2014), UK:<br>Participants (occupational therapists): Female: 57%; Age: Not reported.<br>Burns and Pickens (2017), USA:<br>Participants (home modification professionals): Female: 85%; Mean age: Not reported.<br>Daniel et al. (2013), Switzerland:<br>Participants (patients and occupational therapists): Female: 75.0%; Mean age: 73.0 (patients); Not reported (occupational therapists).<br>Eriksson et al. (2000), Sweden:<br>Participants (occupational therapy students and persons with disability): Female: 56%; Mean age: 26.6.<br>Hamm et al. (2017a, 2017b)a, UK:<br>Participants (older adults and occupational therapists): Older Adults: 70% female; Mean age:	" The availability of new technologies offers potential to improve service delivery; however these technologies are underutilised in clinical practice. Technologies may offer advantages in the conduct of home assessments, especially regarding efficiency, but have not yet been shown to be superior in terms of patient outcome."	**Adverse event/equipment issue**<br>" Patients reported that the videoconferencing equipment was easy to use overall, although 10% experienced some difficulty in understanding the therapist because of an audio delay and 50% experienced difficulty bending low enough to accurately line up the ultrasonic device for measuring some heights, requiring therapist assistance."	 **Cost**<br>"Potential benefits of software for discharge planning identified: reduce cost when transporting client difficult, when the home is some distance away and when the client." (results from one study findings)<br><br>" Cost of assessment: photo-based (77 43 Swiss francs) versus in-home (164 43 Swiss francs)." (included study findings)<br><br>"The results, based on 20 cases, indicated that most issues (79%) were identified using the photographs (1120 environmental factors identified via photos versus 1416 byon-site assessment), and that costs associated with the photography-based assessment were lower."<br><br>" Finally, although telehealth technologies may reduce the occupational therapists travel time, some of the studies have involved

		71.2; Occupational Therapists: 100% female; Mean age: Not reported.<br>Hoenig et al. (2006), USA: Participants (community-dwelling adults): Female: 23.1%; Mean age: 58.2.<br>Hoffmann and Russell (2008), Australia:<br>Participants (patients): Female: 65%; Mean age: 68.<br>Nix and Comans (2017), Australia:<br>Participants (occupational therapists): Female: Not reported; Mean age: Not reported.<br>Palmon et al. (2004), Israel: Participants (occupational therapists): Female: 100%; Age: Not reported.<br>Sanford et al. (2004), USA: Participants (occupational therapists and inpatients): Female: 100%; Age: Not reported.<br>Sim et al. (2015), Australia: Participants (occupational therapists and rehabilitation patients): Female: 50%; Mean age: 72.9.<br>Svarre et al. (2017), Denmark: Participants (app instructors and occupational therapists): Not reported.<br>Threapleton et al. (2017), UK: Participants (occupational therapists and people with stroke): Age: Hospital patients (mean 68 years), Community-			therapists or technicians delivering equipment to the house and teaching the user how to use the technology, offsetting potential time and cost savings."<br><br>**Access**<br><br>"The use of information and communication technologies to assist the process of home assessments offers the potential to increase access for people in rural areas, reduce the costs of home assessments and improve the quality of the process." (comment introduction)

		dwelling survivors (mean 70 years).<br><br>**Gender**<br><br>Atwal et al. (2013), Money et al. (2011)a, UK:<br>Participants (occupational therapists): Female: 80%.<br>Atwal et al. (2014), UK:<br>Participants (occupational therapists): Female: 57%.<br>Burns and Pickens (2017), USA:<br>Participants (home modification professionals): Female: 85%.<br>Daniel et al. (2013), Switzerland:<br>Participants (patients and occupational therapists): Female: 75.0%.<br>Eriksson et al. (2000), Sweden:<br>Participants (occupational therapy students and persons with disability): Female: 56%.<br>Hamm et al. (2017a, 2017b)a, UK:<br>Participants (older adults and occupational therapists): Older Adults: 70% female; Occupational Therapists: 100% female.<br>Hoenig et al. (2006), USA:<br>Participants (community-dwelling adults): Female: 23.1%.<br>Hoffmann and Russell (2008), Australia:			

		Participants (patients): Female: 65%.<br>Nix and Comans (2017), Australia:<br>Participants (occupational therapists): Not reported.<br>Palmon et al. (2004), Israel:<br>Participants (occupational therapists): Female: 100%.<br>Sanford et al. (2004), USA:<br>Participants (occupational therapists and inpatients): Female: 100%.<br>Sim et al. (2015), Australia:<br>Participants (occupational therapists and rehabilitation patients): Female: 50%.<br>Svarre et al. (2017), Denmark:<br>Participants (app instructors and occupational therapists): Not reported.<br>Threapleton et al. (2017), UK:<br>Participants (occupational therapists and people with stroke): Not reported.			
**5**	Nissen (2018)<br>Scoping review<br>N=14*	All fourteen studies involved assessments or interventions aligning with occupational therapy practice. However, only one study featured an occupational therapy practitioner, with social workers, nurses, and psychologists serving as the primary lead health professionals in the others.<br><br>**Age and Gender**<br>NR	Evidence supporting the use of telehealth for delivering OT services to the dementia-caregiver dyad is available. The review encompasses six randomized controlled trials, four one-group pre/post-test studies, and four exploratory/feasibility studies, all exploring telehealth methods for providing services such	NR	**Cost**<br>"Reasons cited for the benefit of using telephone over other technologies was accessibility, ease of use, cost effectiveness, preferred method by older adults, convenience, and ability to remain anonymous in group sessions."

			as assessment, caregiver education, coaching, cognitive behavioral training, counseling, exercise programs, and mantra use. The dementia-caregiver dyad experienced benefits, including a reduction in caregiver burden, depression, stress, anxiety, and hours of care. Additionally, there was an improvement in managing dementia-related behaviors, perceived self-efficacy in caregiving and exercise, caregiver health, self-care skills, exercise adherence, utilization of respite care, and social support. This evidence is valuable for OT practitioners, working as a foundation to support telehealth as an effective method for assisting the dementia-caregiver dyad.		
**6**	Ding<br>(2023)<br>Scoping review<br>N=20	Older people: including people in wheelchairs, stroke, hip fracture, subjective cognitive impairment, mild cognitive impairment, Alzheimer's disease, chronic obstructive pulmonary disease, acquired brain injury, neurocognitive disorder, Parkinson, lung or gastrointestinal cancers.	"The results reveal that telehealth OT has been used successfully for occupational assessment, occupational intervention, rehabilitation counseling, caregiver support, and activity monitoring through multiple mobile	**Adverse events**<br><br>"No serious adverse events were reported in any of the studies we included, so the safety of telehealth OT can be initially confirmed."<br><br>**About caregivers**	**Age**<br><br>"However, the growth rate of healthy life expectancy is significantly lower than that of life expectancy, which means that gerontology is poised to face new opportunities and challenges… These

		**Age**<br><br>Ave. 71.81<br><br>**Gender**<br><br>Not reported	platforms. To the best of our knowledge, this is the first review that combines telehealth and OT in geriatric rehabilitation. Telehealth has extensive potential for use and further development, both during the COVID-19 pandemic and beyond. Further research is needed that will employ large-scale randomized controlled trials to explore new scope and applications for telehealth OT”	"The use of telehealth makes caregiver support more convenient and efficient, and five of the studies we reviewed specifically involved caregiver support (25%)" "Certainly caregivers, as the people most familiar with and connected to the patients, play an especially important role in the daily lives of older people [84–87]. Because telehealth is family centered or community-centered, its advantages may be exploited to the fullest [88–90]."<br><br>**Handling data/data protection/data security**<br><br>"...and nine of the studies (45%) monitored occupational interventions through telehealth platforms that improved security and facilitated OTs’ ability to review and summarize their interventions." (Results)<br><br>**Autonomy**<br><br>“Occupational therapy (OT) is an important component	impairments which occur in later adulthood can reduce their quality of life and well-being, while at the same time increasing the burden on families” (Introduction)<br><br>**Costs**<br><br>“Furthermore, older people have difficulty adhering to traditional rehabilitation because of its high cost, the time spent travelling between rehabilitation facilities and their home, and the safety issues that result from the journey” (Introduction)

				of rehabilitation that promotes functional independence through meaningful occupations and purposeful activities to enhance occupational performance.” (Introduction)<br><br>“The focus of OT services is to improve a person's independence and a client's quality of life. OT practitioners supported by the philosophy of profession can work with clients of all ages, abilities and in many different settings.” (Discussion)	
**7**	Giustiniani (2023)<br>SR<br>N=16	Breast cancer<br><br>**Age**<br>C: 49.76<br>T: 53.40<br><br>**Gender**<br><br>100% Women	In the 2 studies including OT + tele:<br>“Improvement in QoL, maintained after 6 months”<br><br>“Greater improvements in selective attention, working memory, and processing speed, anxiety, and functional capacity at 8 weeks and 6 months. Fatigue perception and pain were also improved”<br><br>Study conclusion:<br>“Similarly, with respect to psychological	**Adverse events**<br><br>Not mentioned	**Gender**<br><br>“ The number of women diagnosed with breast cancer is dramatically increasing, and survivors must face many challenges while they continue with their lives during and after cancer treatment.”<br><br>**Cost / access**<br><br>“These technologies offer the opportunity to overcome the patient’s mobility problems and

			concerns, novel telemedicine-based approaches are needed that focus on specific interventions related to the wide range of difficulties experienced by breast cancer patients, namely, depression and anxiety, and the patients' demographics should be the focus of new RCT studies. Along these lines, further studies should target both cognitive and psychological factors with specific telemedicine-based protocols that also consider the molecular classification and new standard of therapy."		reduce costs for the national health system. To date, telemedicine has been widely used with promising results in terms of cost-effectiveness, in mental health, and in cognitive impairment" (Introduction)<br><br>"Indeed, these programs also provide the opportunity to access rehabilitation for patients who cannot reach care centers, allow for the continuity of care over time and space, reduce care costs, and improve comfort for the patients, thus reducing the drop-out rates" (Introduction)
**8**	Hayes (2023)<br><br>Scoping review<br>N= 117<br><br>Note: 19% of studies about telehealth / 17% about disabilities / 12% neurological insult / …	"The clinical population most served were people who experienced an injury (21%), particularly people who experienced a neurological insult (stroke, traumatic brain injury, and spinal cord injury) (13%). Developmental diagnoses were the next most discussed group (20%), followed by clinical populations with illnesses such as cancer or dementia (17%)."	"Despite demonstrable global need in non-urban spaces, this review demonstrates that occupational therapy has engaged in proportionally little research regarding nonurban practice. Further research is required to identify effective approaches to non-urban service delivery, the level of contextual analysis and understanding which can	**Adverse events**<br><br>Not mentioned	**General inequity / Urban – rural**<br><br>"Globally, regional, rural, and remote (henceforth referred to as non-urban) people experience higher mortality, morbidity, and disability rates, while having less access to health care, professionals, and funding (Scheil Adlung, 2015; World Health Organisation, 2021). This non-urban inequity extends across countries, regardless of

			be provided by urban-based occupational therapists to non-urban contexts, and how occupational therapists can implement community level enabling strategies to help reduce inequity between urban and non-urban populations”		development level, and amplifies existing inequities such as race, gender, and social class, rather than being explained by it (Taylor, 2019). For example, within the same country, non-urban women are generally poorer and sicker than urban women; non-urban people of colour are generally poorer and sicker than urban-based people of colour (Taylor, 2019). The global occupational therapy community stated its commitment to promoting occupational justice and human rights (World Federation of Occupational Therapists, 2019), including the right to an adequate standard of living and health care services for all people (United Nations, 1948). The inequity between urban/non-urban populations within and between countries is therefore of interest to occupational therapists.” (Introduction)<br><br>*Note: the article is about the services in non-urban settings with a Global North and South approaches*.

					"Key Points for Occupational Therapy • Despite community level urban/rural inequity, the most common non-urban services described used an individualist rehabilitative approach • Non-urban services are frequently provided by urban-based occupational therapists via outreach which may limit contextual understanding • Further research is required on how occupational therapy can address rural/urban inequity" (Framed comment)<br><br>"Occupational therapy is now present in 98 countries (World Federation of Occupational Therapists, 2021), but fewer than 20% of these countries have published anything about non-urban occupational therapy services over the decade studied in the databases searched. This limited engagement with non-urban research is consistent with existing

					literature that non-urban health research receives both less attention and funding than in urban spaces (Barclay et al., 2018); however, by not focusing on nonurban practice, occupational therapy risks neglecting the health and wellbeing of nearly half the world's population, who in nearly all cases, have greater need than those in urban populations. Non-urban research scarcity not only limits health access now but may also decrease the visibility and thereby perceived value of and interest in rural practice for future therapists (Malatzky & Bourke, 2016). Limited non-urban research contributes to the construction of non-urban practice as less visible, legitimate, and valued and may tacitly reinforce and reproduce limits on non-urban research/practice interest, further heightening non-urban inequity (Malatzky & Bourke, 2016)." (Discussion)<br><br>Further research is required regarding how occupational therapy can

					best serve non-urban people. Most occupational therapy publications about occupational therapy services appear stratified by age groups and diagnostic presentations, for example, children with autism (Johnsson et al., 2019; Little et al., 2018; Little & Wallisch, 2019; McClure et al., 2010; Monz et al., 2019) or adults with stroke (Danzl et al., 2013, 2016; Hermann et al., 2010; Marsden et al., 2010; Merchant et al., 2016). However, stratifying by diagnostic and age groups underestimates contextual and environmental impacts (Taylor, 2019) and may limit access for people who do not fit neatly into these classifications, possibly fundamentally misunderstanding non-urban contexts. (Discussion)<br><br>"The inequity in healthcare access, formal education access, and income are population level issues impacting on health outcomes and the context of practice (Taylor, 2019). Misunderstanding the

					context in which clients are working may result in poor occupational therapy theory application and potentially reduce client trust, rapport, and belief in the usefulness of therapy (Dew et al., 2013). (Discussion)<br><br>"We identified very few publications using community level enablement strategies. Only four publications addressed redistributive justice, and all but one of these were from the Global South (Costa, 2012; Lauckner & Stadnyk, 2014; McAdam & Rose, 2020; Watson & Duncan, 2010). Most of the Global North community level strategies were related to transformation, service development regarding non-urban service uptake/relevance to increase participation in individualised therapy (e.g. Churchill, 2016; Curtis, 2012; Gauld et al., 2011a, 2011b; Keightley et al., 2011; Kitzman & Hunter, 2011; Morgan et al., 2019). Given the inequity experienced by non-urban communities (Taylor, 2019), redistributive

					justice, focusing on increased access to resources and opportunities, may be more impactful in rural communities than any individualist strategy (Watson, 2013). Further research should consider how occupational therapists can engage with and implement community level enablement strategies in non-urban spaces." (Discussion)
**9**	Suder<br>(2023)<br>SR<br>N=23	Pediatric Chronic Pain / Juvenile arthritis<br><br>**Age**<br>Two reviewed studies about tele: 12 to 18 years<br>Ave.14.8 years<br>For all 23 studies: "It is noted that the mean age across studies was from 9.9 to 16 years of age, indicating that the participants for these studies were older children and adolescents"<br><br>**Gender**<br>Not specified	**Adverse events**<br><br>Not mentioned<br><br>1st study: "No significant difference between groups in pain or function (p > .05)"<br><br>2nd study: "difference in pain scores for intervention group (p = .03)<br><br>(Note: there are 3 studies about virtual technologies, but only 2 are real tele)<br>"The internet-based studies focused on technological intervention methods and provide inconclusive evidence. Two of the	**Autonomy**<br><br>"Internet-based studies evaluated virtual or technological methods to deliver intervention sessions … or audio recordings … The internet-based programs evaluated the feasibility and effectiveness of virtual self-management interventions with social support for adolescents (Kohut et al., 2016; Stinson et al., 2010). No significant improvements in functional ability such as social participation and medical self-management were found." (Results)<br><br>"Existing studies on pediatric pain	**Gender**<br><br>"Participants were largely Caucasian females. Therefore, interventions are not necessarily generalizable to males or other races, and cultural and gender-related differences may strongly influence participation and reported outcomes" (Limitations)

			three articles displayed a significant decrease in pain frequency (Stinson et al., 2010; Van Tilburg et al., 2009), whereas only one demonstrated a reduction in disability (Van Tilburg et al., 2009). Treatment adherence to intervention is difficult to monitor secondary to lack of face-to-face contact. However, utilization of telehealth continues to increase and therefore more efficacy research is warranted. Virtual formats may be effective; however, better intervention guidelines for occupational therapy practice would be useful to determine how to effectively decrease both pain and disability" (Discussion)<br><br>"Gaps in the existing literature were found, with few studies including occupational therapists in the delivery of interventions despite many approaches falling within their scope of practice … Occupational therapists can also	interventions, while not all derived from occupational therapy literature, may still be of interest to guide physical, contextual, and psychological interventions for improving participation in functional activities. The Occupational Adaptation Model (Schkade & Schultz, 1992) suggests clients need to be able to adapt to their environment to participate in meaningful occupations, while the Fear Avoidance Model of Chronic Pain highlights fear of experiencing pain limits activity participation (Vlaeyen & Linton, 2000). Occupational therapists uniquely incorporate adaptive strategies to minimize pain and increase pain tolerance to maximize participation and independence." (Discussion)	

			incorporate their understanding of emotional, cognitive, and sensory influences on pain to provide coping strategies. These strategies may be most effective within an interprofessional care team, reinforcing the need to advocate for the inclusion of occupational therapy. In addition, it is necessary to derive more rigorous intervention studies using randomized control groups to address pain and functional disability to promote occupational therapy's role in this field."		

Note: BTCs=Behavior Change Techniques; CBT= cognitive behavioral therapy; MS= multiple sclerosis; MSK=Musculoskeletal; OT=Occupational therapy; SR= systematic review; TBI=Traumatic brain injury; TR= Telerehabilitation; UR=umbrella review

*= A single study specifically featured an occupational therapist as the primary service provider, while the remaining 13 studies concentrated on services falling within the purview of occupational therapy practice.
