# Supplementary material for: A Rapid Review of Ethical and Equity Dimensions in Telerehabilitation for Physiotherapy and Occupational Therapy
Source: Int J Environ Res Public Health. 2025 Jul 9;22(7):1091. doi: 10.3390/ijerph22071091 (PMC12294586; doi:10.3390/ijerph22071091)
Supplement: Supplementary file 1 [file ijerph-22-01091-s001.zip › Table S4- Ethics Themes and Illustrative Examples in Included Studies April 10.pdf]

Table S4: Ethics Themes and Illustrative Examples in Included Studies

Ethics themes (Ethical Principle)	Examples of concerns
**Adverse events (Non-maleficence)- actual and potential risk**	*"The current data are encouraging and support continuity of rehabilitation care through ICTs, but the quality of primary research has to be improved dramatically to have a clearer picture of benefits and risks associated with assisting patients at a distance, once discharged at home." (Agostini, 2015)*[24]<br><br>*"Authors defined serious event according to FDA. Exercise therapy appears safe and does not increase the risk of MS relapse or adverse events. It can reduce self-reported fatigue for people with MS and can be prescribed without harm. However, there's no clear evidence regarding the best type, duration, intensity, or frequency of exercise due to study diversity." (Amatya, 2019)*[25]<br><br>*"Low image resolution due to poor bandwidth has made it difficult for the TR physiotherapist to guide the patient to palpate the anatomical location of the source of pain and tenderness. This barrier may be overcome by providing a body chart via the TR system ahead of time. In addition, the validity of SOTs and NDTs via the TR method might be improved by guiding and training the patients or caregiver through real-time feedback, supplemented by high-quality video or a video weblink. Poor rapport during the TR session may have a negative influence on clinical reasoning in the diagnosis of MSDs via TR." (Mani, 2016)*[50]
**Autonomy (Respect for autonomy)**	*"Participants reported a sense of control over their rehabilitation and appreciated the flexibility of scheduling the day and time to exercise. Participants demonstrated significant increase in self-management and functional measures and reduction in time on tasks." (Chen, 2019)*[27]<br><br>*"Four studies measured static muscle strength by applying patients' self-resistance and patients were asked to perform modified self-administered SOTs under the guidance of the TR physiotherapist. Self-palpation was also used in one study for pain assessment." (Mani, 2016)*[50]<br><br>*"Self-management of chronic/long-term illnesses through education and supportive interventions can not only decrease utilization of health care services but may also lead to improvements in clinical outcomes and overall quality of life." (Slattery, 2019)*[37]
**Privacy (Respect for Privacy, Confidentiality and data security)**	*"The concerns surrounding the [...] security of patient data are one of the main problems inhibiting the introduction of e-health services in Australia. At present, desktop videoconferencing programs that offer secure video rooms are available at a low cost ($35/month plus 6¢/min). The security is provided by using 128-bit AES encryption for data in transit and unique URLs for each of the online meetings to ensure that only authorized people are able*

	to access the Web conferences. Finally, an alternative way of ensuring patient data security is to avoid sending sensitive information over the videoconferencing network." (Pietrzak, 2013)[57]<br><br>*"Future developments should focus on adequate data storage systems and real-time analysis of continuous updated information, to provide immediate feedback to patients. Those systems should also guarantee privacy protection." (Berton, 2020)*[33]<br><br>*Concerns have also been raised about the security of data transfer and how patient confidentiality can be maintained (American Telemedicine Association 2010)." (Laver, 2020)*[30]<br><br>*Further research is needed into information privacy, how ehealth supports continuity of care and promotion of client-clinician relationships. (Iacono, 2016)*[56]
