# Supplementary material for: A Rapid Review of Ethical and Equity Dimensions in Telerehabilitation for Physiotherapy and Occupational Therapy
Source: Int J Environ Res Public Health. 2025 Jul 9;22(7):1091. doi: 10.3390/ijerph22071091 (PMC12294586; doi:10.3390/ijerph22071091)
Supplement: Supplementary file 1 [file ijerph-22-01091-s001.zip › Table S5 Equity themes and illustrative examples in included studies April 10.pdf]

Table S5: Equity themes and illustrative examples in included studies

Equity themes (PROGRESS Plus reference/MERTH Framework)	Examples of concerns
**Access**	***Access***<br><br>*Rochette 2013 study Recruited from 11 acute care hospitals located in urban and rural areas across 4 Canadian provinces. "People in rural and remote areas are unlikely to have access to rehabilitation teams with expertise in stroke, and they may not have access to rehabilitation clinicians at all. Eliminating the need for travel to rehabilitation centres may also benefit people with severely restricted mobility who have difficulty travelling or are unable to travel. Telerehabilitation is also likely to be beneficial in low-resource settings where access to health professionals is poor but access to devices such as mobile phones is present." (Laver, 2020)[25]*<br><br>*"Having rapid and remote access to a health professional for advice and tailored support has been reported in previous studies of Internet-based interventions for care recipients as the primary factor predicting adherence" .... "Knowing that they have access at any time and place to professional support may also make caregivers feel less worried." (Guay, 2017)[27]*<br><br>*"This system is advantageous not only for those who live far from rehabilitation centers but also for people with severe disabilities as moving is not necessary." (Berton, 2020)[24]*
**Gender**	***Gender bias***<br><br>*"Gender bias towards men (more male participants than women)." (Appleby, 2019)[28]*<br><br>*"Of the studies that reported on the gender of participants (15 studies), all studies except 3 had a greater number of female participants." (Hewitt, 2020)[29]*<br><br>*"When the results were stratified by sex, men in the intervention group were less likely than women to receive appropriate care as15% and 44% respectively." (Yadav, 2019)[30]*
**Cost**	*"Internet-based interventions can thus offer an easily accessible alternative and can be more cost-effective than traditional face-to-face interventions. asynchronous communication can cost less to developers and be used more easily by caregivers than complex synchronous communication modes, such as videoconference. Isolation of the effects of each component could help future research provide better cost-benefit analyses because some components require more resources than others to develop." (Guay, 2017)[27]*

	"TR also includes tele-evaluation in OT, such as the use of a low-cost traditional telephone system for conducting phone interviews as an alternative for cognitive assessment, wheelchair and assistive device prescription and home modification, etc." (HungKn, 2019)[31]<br><br>*"There is limited data on safety, process evaluation and no data on cost-effectiveness of telerehabilitation."*<br>*"There was no evidence of these programmes' cost-effectiveness. No studies reported any data on cost-effectiveness, investment costs or resource utilization." (Amatya, 2015)*[32]
**Digital Divide**	***Internet access***<br><br>*"A high level of access to the internet amongst Australians demonstrates a vector through which the benefits of ehealth for rural and remote areas can be realised, particularly assisted by the National Broadband Network (NBN). Unfortunately, the specific mechanisms and nature of the infrastructure have been disputed across governments, resulting in delays in the roll out and inequalities in access." (Iacono, 2016)*[26]<br><br>***Digital Literacy***<br><br>*"Clinicians and patients may not possess the technical expertise to establish systems and to troubleshoot information and communication technologies. It has been recommended that service providers ensure that technical requirements are met (such as having adequate bandwidth), provide access to technical support and provide training to all users (clinicians and patients)." (Laver, 2020)*[25]<br><br>*"It is worth noting that participants' computer literacy level has no effect on acceptability or on the utilization of the tele-pulmonary rehabilitation systems." (Almojaibel, 2016)*[33]
